# Phage-antibiotic synergy suppresses resistance emergence of *Klebsiella pneumoniae* by altering the evolutionary fitness

**DOI:** 10.1128/mbio.01393-24

**Published:** 2024-09-09

**Authors:** Kunhao Qin, Xing Shi, Kai Yang, Qiuqing Xu, Fuxing Wang, Senxiong Chen, Tingting Xu, Jinquan Liu, Wangrong Wen, Rongchang Chen, Zheng Liu, Li Cui, Kai Zhou

**Affiliations:** 1 Department of Pathogen Biology, Shenzhen University Medical School, Shenzhen, China; 2 Shenzhen Institute of Respiratory Diseases, Shenzhen People's Hospital (The Second Clinical Medical College, Jinan University; The First Affiliated Hospital, Southern University of Science and Technology), Shenzhen, China; 3 Jiangxi Province Key Laboratory of Organ Development and Epigenetics, Clinical Medical Research Center, Affiliated Hospital of Jinggangshan University, Health Science Center, Medical Department of Jinggangshan University, Ji'an, China; 4 Key Laboratory of Urban Environment and Health, Fujian Key Laboratory of Watershed Ecology, Institute of Urban Environment, Chinese Academy of Sciences, Xiamen, China; 5 Kobilka Institute of Innovative Drug Discovery, School of Medicine, Chinese University of Hong Kong, Shenzhen, China; 6 Clinical Laboratory, The Affiliated Shunde Hospital of Jinan University, Foshan, China; 7 Clinical Laboratory Centre, The First Affiliated Hospital of Jinan University, Guangzhou, China; Emory University, Atlanta, Georgia, USA

**Keywords:** hypervirulent *Klebsiella pneumoniae*, phage-antibiotic synergy, evolutionary trajectory, metabolic activity

## Abstract

Phage-antibiotic synergy (PAS) represents a superior treatment strategy for pathogen infections with less probability of resistance development. Here, we aim to understand the molecular mechanism by which PAS suppresses resistance in terms of population evolution. A novel hypervirulent *Klebsiella pneumoniae* (KP) phage H5 was genetically and structurally characterized. The combination of H5 and ceftazidime (CAZ) showed a robust synergistic effect in suppressing resistance emergence. Single-cell Raman analysis showed that the phage-CAZ combination suppressed bacterial metabolic activities, contrasting with the upregulation observed with phage alone. The altered population evolutionary trajectory was found to be responsible for the contrasting metabolic activities under different selective pressures, resulting in pleiotropic effects. A pre-existing *wcaJ* point mutation (*wcaJ_G949A_*) was exclusively selected by H5, conferring a fitness advantage and up-regulated activity of carbohydrate metabolism, but also causing a trade-off between phage resistance and collateral sensitivity to CAZ. The *wcaJ* point mutation was counter-selected by H5-CAZ, inducing various mutations in *galU* that imposed evolutionary disadvantages with higher fitness costs, and suppressed carbohydrate metabolic activity. H5 and H5-CAZ treatments resulted in opposite effects on the transcriptional activity of the phosphotransferase system and the ascorbate and aldarate metabolism pathway, suggesting potential targets for phage resistance suppression. Our study reveals a novel mechanism of resistance suppression by PAS, highlighting how the complexity of bacterial adaptation to selective pressures drives treatment outcomes.

**IMPORTANCE:**

Phage-antibiotic synergy (PAS) has been recently proposed as a superior strategy for the treatment of multidrug-resistant pathogens to effectively reduce bacterial load and slow down both phage and antibiotic resistance. However, the underlying mechanisms of resistance suppression by PAS have been poorly and rarely been studied. In this study, we tried to understand how PAS suppresses the emergence of resistance using a hypervirulent *Klebsiella pneumoniae* (KP) strain and a novel phage H5 in combination with ceftazidime (CAZ) as a model. Our study reveals a novel mechanism by which PAS drives altered evolutionary trajectory of bacterial populations, leading to suppressed emergence of resistance. The findings advance our understanding of how PAS suppresses the emergence of resistance, and are imperative for optimizing the efficacy of phage-antibiotic therapy to further improve clinical outcomes.

## INTRODUCTION

*Klebsiella pneumoniae* (KP) has become one of the major causes of hospital- and community-acquired infections worldwide (1, 2). According to the virulence phenotype, KP is categorized into classical and hypervirulent populations. Classical KP (cKP) is typically a problem among neonates, elderly, and immunocompromised individuals in the healthcare setting (3), while hypervirulent KP (hvKP) can cause invasive infections in healthy individuals in the community (4, 5). Antibiotic resistance is frequently associated with cKP but not hvKP, however, recently there have been increasing reports of hvKP strains that produce Extended Spectrum Beta-lactamases or even carbapenemases (2, 6, 7). Such a convergence immensely worsens the clinical outcome of infections. Therefore, there is an urgent need to develop efficient therapeutic approaches to tackle this problem.

Phages have recently received renewed attention as alternative antibacterial agents (8–10), and have been used for therapeutic and prophylactic purposes with less adverse effect on normal microbial flora and minimal side effects (11, 12). Although promising results have been reported in numerous studies, the rapid development of phage resistance by pathogens leads to widespread skepticism about their true therapeutic efficacy (13, 14). In such cases, combination therapy of phages and antibiotics has been increasingly proposed as a promising strategy. It has been shown that sub-inhibitory concentrations of antibiotics can enhance phage productivity to improve phage-mediated bacterial elimination, a phenomenon termed phage-antibiotic synergy (PAS) (15). The significant advantage of this approach lies in its potential to slow down resistance evolution and steer it in a favorable direction with a more robust suppression of bacteria (16–18). Several mechanisms of PAS have been proposed, mainly based on phenotypic studies, including cell elongation/filamentation by antibiotics and antibiotic-induced phage production (19). However, the molecular mechanism of resistance suppression by PAS remains largely unknown.

In this study, we aim to investigate the molecular mechanism of resistance suppression by a phage-antibiotic combination in KP. In particular, we conducted the study at both population-level and single-cell levels using multi-omic techniques and a Raman platform to systematically investigate the impact of PAS on biological processes and evolutionary dynamics of hvKP. The key genetic alternations responsible for resistance suppression by PAS were identified. The study demonstrates a novel mechanism of resistance suppression by a phage-antibiotic combination in KP.

## MATERIALS AND METHODS

### Bacterial strains and culture conditions

Thirteen clinical isolates of KP were obtained from our previous study (20). Three type strains (hvKP ATCC 43816, *Escherichia coli* MG1655, and *Staphylococcus aureus* ATCC 25923) were purchased from the American Type Culture Collection (ATCC). All strains were grown in Luria-Bertani (LB) medium at 37°C. The bacterial strains used in this study are listed in Table S1.

### Bacteriophage isolation and propagation

Phage isolation, propagation, and purification were performed as previously described (21). hvKP ATCC 43816 (wild type, WT) was used as a host for phage isolation from hospital wastewater. Sewage samples were filtered using 0.22 µm pore size filters (Millipore, Billerica, MA, USA) to remove most contaminants. The purified phages were stored at 4°C.

### The characterization of phage H5

Phage host range was determined using a spot test and efficiency of plating (EOP) method in combination with double-layer agar plate methods as previously described (22). pH and thermal stability of phage H5 were evaluated as reported (23). To determine the optimal multiplicity of infection (MOI), bacteria cultured in log phase (OD_600_ = 0.8, approximately 10^8^ colony-forming units (CFU)/mL) were infected with H5 at nine different MOIs ranging from 10 to 0.0000001 [approximately 10^9^ to 10^1^ plaque-forming units (PFU)/mL], and the number of phage progeny was counted after 3-h incubation. H5 adsorption kinetics and one-step growth experiment at the optimal MOI (0.00001) were performed as previously described (24). Lysis kinetics for H5 was measured using the plate reader Biotek EPOCH2 (Biotek, Winooski, VT, USA), with OD_600_ measured every 10 min for a total of 24 h with shaking at 360 rpm.

### Transmission electron microscopy

A high-titer phage lysate was subject to ultra-centrifugation for purification using CsCl. A portion of the purified phages was applied to a carbon-coated grid, CF300-Cu grid (EMS, Hatfield, PA, USA) set down for 90 s and then stained with 2% uranyl acetate for 60 s and washed with PBS twice. The grid was examined in a Talos 120C transmission electron microscope (TEM) (Thermo Fisher Scientific, Waltham, MA, USA) at 120 kV, the images were captured using a Ceta camera (Thermo Fisher Scientific, Waltham, MA, USA) with magnification of ×92,000.

### Electron cryo-microscopy and image processing

An aliquot of 2.5 µL of purified H5 phage at the concentration 10^10^ PFU/mL was applied to a 300 mesh Quantifoil R 1.2/1.3 copper grid (hole size 1.2 µm) (Quantifoil Micro Tools, Jena, Germany) and rapidly snap frozen in liquid ethane using an FEI Vitrobot. A total of eight grids were used, including two grids with a continuous carbon film underneath the samples. Cryo-EM images were collected in a TITAN KRIOS cryo-electron microscopy (Thermo Fisher Scientific, MA, USA) operated at 300 keV and liquid nitrogen sample temperature. The microscope is equipped with a K3 direct electron detector and an energy filter (20 eV slit width used for data collection) (Gatan, Pleasanton, CA, USA). Approximately 6,300 movies were acquired at a detector magnification of ×81,000 (1.06 Å/pixel sampling rate) with a defocus range of 0.5–2.5 μm.

Raw movie frames were aligned using a 9 × 7 patch in MotionCor2, and contrast transfer function (CTF) parameters were estimated using Gctf and CTF in JSPR (25). Only the micrographs with consistent CTF values, including defocus and astigmatism parameters, were retained for subsequent image processing in a hierarchical way (26). This process retained 1,298 micrographs from 6,300 raw movies. Templates for particle selection were created by projecting the 3D volume of the T7 phage (27, 28). The 48,775 particles picked by the template selected were subjected to two rounds of 2D classification, reducing their size to 25,749, and then reducing to 20,346 by 3D classification in Relion. In cryoSPARC (29), after several rounds of ab initio refinement, the particles retained at 18,864 were subjected to non-uniform refinement for a 3.14 Å reconstruction. Image parameters were converted back to Relion and cryoSPARC using the pyem package.

The T7 phage model PDB id:3j7x (28) was used as the starting model. It was then manually mutated and fitted to the reconstructed density map. The atomic model of the large ribosomal subunit was refined in real space using PHENIX (30).

### Antimicrobial susceptibility testing

The antibiotics used in this study were purchased from Meilunbio (Dalian, China). Each antibiotic was dissolved in distilled water and filter sterilized. Trimethoprim and rifampicin were dissolved in dimethyl sulfoxide. Chloramphenicol was dissolved in absolute ethanol. Antimicrobial susceptibility testing was performed by the agar dilution and/or broth microdilution methods. The minimum inhibitory concentrations (MICs) of the antibiotics were evaluated according to the Clinical and Laboratory Standards Institute guidelines (M100-S30, 2020) (31).

### Phage-antibiotic synergy testing

Different types of antibiotics were tested to measure the synergistic relationship with H5, including an RNA synthesis inhibitor (rifampicin), a cell membrane disruptor (polymyxin B), a folic acid synthesis inhibitor (trimethoprim), protein synthesis inhibitors (kanamycin and chloramphenicol), DNA topoisomerase inhibitors (ciprofloxacin and levofloxacin), and cell wall synthesis inhibitors [ceftazidime (CAZ) and meropenem]. A subculture of ATCC 43816 was incubated to log-phase (OD_600_ = 0.8, approximately 10^8^ CFU/mL), and 100 µL was inoculated into each well of the microtiter plate that contained the checkerboard of phage and antibiotic concentrations. PAS testing was performed using a Biotek EPOCH2 and OD_600_ was measured every 10 min for a total of 24 h with shaking at 360 rpm as previously described (32). Here, we assessed bacterial growth when exposed to different concentrations of antibiotics that blanket the MIC over the MOI of the phage titer over time. Absorbance was read as a stand-alone parameter and converted into a heat map representing the percentage reduction in bacterial population, known as a synogram. This was used to measure Fractional inhibitory concentration (FIC) indices, and the results were interpreted as synergistic if FIC ≤ 0.5 and there was a 90% or greater reduction in bacterial density (32–34). To further evaluate the synergistic effect of H5 and CAZ, the effects of CAZ on phage characteristics (one-step growth curve, lysis time, burst size, and gene expression level) and cell morphology were measured (35).

### Luria–Delbrück fluctuation tests

Luria–Delbrück fluctuation tests were performed as previously reported (36, 37). Briefly, overnight cultures were 100-fold diluted into LB cultures with 220 rpm agitation at 37°C to reach the OD of 0.3 (~10^8^ CFU/mL). The cultures were further diluted to a final concentration of 10^3^ CFU/mL. Aliquots of 1 mL of dilution were inoculated into 10 tubes (group A), and aliquots of 10 mL bacterial dilutions were inoculated into one tube (group B). All tubes were incubated at 37°C with shaking until the OD reached 0.6–0.8. Phage-resistant colonies were enumerated after 24 h incubation on LB agar plates containing phage (MOI = 1,000) at 37°C. For group B, 10 technical replicates were sampled from the same tube. One colony was randomly selected from each plate and submitted to whole-genome sequencing (WGS) (20 mutants in total).

### Mutant construction and complementation

The primers used for mutant construction are listed in Table S7. Mutants were constructed using the suicide vector pRE112 (38), and the gene spacer DNA fragment was cloned into a pSGKP-km vector. The synthetic oligonucleotide for each homology extension of the target gene was used as a donor template. Both the resulting plasmids and the donor template DNA were transformed into pCasKP-harboring ATCC 43816 by electroporation, and the integration was selected on LB plates containing 5% sucrose at 37°C. For the complementation, genes and their promoter regions were amplified by PCR and cloned into pBAD33 for complementation, and the resulting plasmids were introduced into mutants via electroporation. PCR and DNA sequencing were used to confirm the final constructions.

### Competition assays and calculation of epistasis

Competition assays were performed in LB media as previously described (8). The relative fitness of the two strains was determined by the competition index (CI), which was calculated as the output/input ratio of the competitor compared to the reference strain (39). If CI > 1, it indicates that the fitness of resistant bacteria is stronger than that of sensitive bacteria; if CI < 1, it indicates that the fitness of resistant bacteria is weaker than that of sensitive bacteria; and if CI = 1, it indicates that the competitiveness of the two is equal.

For any quantitative trait, epistasis (e) can be calculated as e = W_AB_ × W_ab_ − W_Ab_ × W_aB_, where W_AB_ is the value of the trait in the wild type, ab is the double mutants, and the single mutants are represented as Ab and aB (40). Negative epistasis (e < 0) occurs when the value of the trait is lower in the double mutant (i.e., ∆*wcaJ* with the duplication) than would be expected from the single mutant (i.e., ∆*wcaJ* or the duplication), and vice versa for positive epistasis (e > 0).

### Raman spectral measurement and data analysis

The mutants and the wild type were exposed to different treatments and incubated in LB broth amended with 50% D_2_O (vol/vol, 99 atom% D, Merck) at 37°C for 24 h. Bacterial cells were harvested by centrifugation at 6,000 rpm for 3 min. The cell pellets were then washed twice with sterile water to remove residual media. An aliquot of 3 µL samples was spotted onto an aluminum (Al) foil substrate (41) and dried at room temperature prior to Raman measurements. Raman spectra were acquired using a LabRAM Aramis confocal Raman system (HORIBA Jobin-Yvon, Japan) equipped with a 532 nm Nd:YAG excitation laser and a 300 grooves/mm diffraction grating. A 100× dry objective (N.A. 0.9, Olympus, Japan) was used for sample observation and Raman signal acquisition. Raman signals were detected by a charge-coupled device cooling down to −70°C. The laser power for each sampling point was about 3.5 mW. The acquisition time for each spectrum was 25 s, and at least 100 spectra were acquired for each treatment with a spectral resolution of 5.3 cm^−1^.

Raman spectra were analyzed as previously described (42). Briefly, all raw spectra were pre-processed for baseline correction using Labspec5 software (HORIBA Jobin-Yvon, Japan). The spectral wavenumbers were calibrated by aligning with the phenylalanine peak at 1,004 cm^−1^ as standard via a customized script. Vector normalization was conducted in the Matlab (version R2020b) environment using the IRootLab toolbox. CD ratios of CD/(CD + CH) were calculated by integrating the CD band (2,040–2,300 cm^−1^) and CH band (2,800–3,100 cm^−1^). For Raman peaks in the fingerprint region, according to the previously reported method (43), the maximum intensity of each peak and the intensity of two neighboring channels were added to obtain the relative intensity values.

### Sequence-structure analysis and structural modeling of protein

Structure-based sequence alignments, generated using MAFFT (44) as implemented in ConSurf (https://consurf.tau.ac.il/consurf_index.php) (45), were rendered with ESPript (46). For calculating consensus information at different thresholds, a ConSurf alignment that sampled homologs with 35%–95% identities were first pruned of incomplete sequences (yielding a final set of 150 aligned sequences) and amino acid site conservation scores were calculated using Rate4Site (47).

The amino acid sequences of WcaJ were submitted to AlphaFold2 v2.3.2 (48) to construct 3D structures. The root-mean-square deviation (RMSD) of wild type and variants was calculated using PyMol v2.5.0 (http://www.pymol.org/pymol). Overlays of these structures and protein-molecule docking were generated using AutoDock Vina v2.0 (49). Hydrogenated WcaJ and UDP-D-glucose were used to perform flexible ligand docking in AutoDock Vina with default parameters to predict the binding sites between the protein and UDP-D-glucose.

### *Galleria mellonella* larvae infection model

The virulence was measured using *G. mellonella* larvae as an infection model (50). *G. mellonella* larvae, 2.0 to 2.5 cm in length, were injected with serial dilutions of bacterial suspension (25 µL/larvae) into the left hind proleg using a 29-gauge insulin needle (Becton, Dickinson and Company, Franklin Lakes, NJ, USA). For each strain, five dilutions were made and each dilution was injected into a group of 10 larvae for three biological replicates. Control larvae were injected with PBS only. Larvae were then incubated at 37°C in the dark and monitored for survival for up to 72 h.

### WGS and data analysis

Phage genomic DNA was prepared using a Viral DNA Kit (Omega, Georgia, USA), and was sequenced on an Illumina HiSeq 2500 sequencer (Novogene, Beijing, China). For quality control, the raw reads were trimmed using Trimmomatic v0.39 with “LEADING: 3 TRAILING: 3 SLIDINGWINDOW: 4:20 MINLEN: 60” (51). The high-quality reads were assembled into contigs using SPAdes v3.15.5 with “--careful -k 21,33,55,77 --phred-offset 33” (52). The completeness and quality of the assembled scaffolds were assessed using VIBRANT v1.2.0 with default settings to confirm the contig as a high-quality draft (53). The genome termini and packaging mechanism were identified using PhageTerm v1.0.12 with default settings (54). Gene prediction of the reorganized H5 gnome was performed using prokka 1.14.6 (55) and VIBRANT v1.2.0, followed by manual curation with BLAST at NCBI against the non-redundant protein sequence database. Comparative genomic analysis was performed by selecting the top two genomes using megablast at NCBI and visualizing the results using EasyFig v2.2.2 (56).

Genomic DNA of bacteria was extracted using a FastPure Bacteria DNA Isolation Mini Kit (Vazyme, Nanjing, China) and subjected to WGS on an Illumina novaseq 6000 system (Illumina, San Diego, USA) to obtain 150 bp paired-end reads. After trimming using Trimmomatic, the high-quality reads were mapped to the reference genome using Bowtie2 v2.2.5 with “--no-mixed --no-discordant --very-sensitive-local” (57). Following the alignment, PCR duplicates were removed using Picard MarkDuplicates v2.26.0 (http://broadinstitute.github.io/picard/). Single nucleotide polymorphisms (SNPs) and INDELs (insertions/deletions) were detected using BCFtools v1.14 (58), with stringent pre-filtering for quality, depth, and mapping scores (QUAL > 30, DP > 5, and MQ > 20). SNPs and INDEL calls with less than 70% of supporting reads were excluded to further ensure the reliability of the variant calls. The detected SNPs and INDELs were checked and validated using CLUSTAL v2.1 (59) and Sanger sequencing. SNP annotation was performed using SnpSift v5.1d (60), enriching the identified variants with detailed genes and effects.

### RNA-seq and data analysis

Bacterial isolate was cultured to log-phase (OD_600_ = 0.8, approximately 10^8^ CFU/mL), then the precipitate was collected by centrifugation at 11,000 *× g* (2 min) for RNA extraction. The quality of the RNA samples was examined using the Bioanalyzer 2100 system (Agilent Technologies, CA, USA). RNA-seq library preparation and sequencing were performed as previously described (61).

Raw reads were trimmed using Trimmomatic v0.39 and then mapped to the reference genome ATCC 43816 (NZ_CP064352) using Bowtie2 v2.2.5 (57). Gene expression levels were quantified by calculating read counts with featureCounts v2.0.0 using default parameters (62), followed by the identification of differentially expressed genes with edgeR v3.32.1 (63). The low-expressed genes were filtered out using filterByExpr and then normalized using the trimmed mean of the M-values method in calcNormFactors, and differential expression was statistically tested using glmQLFTest function (64). Genes with fold change > 2 and *P*-value < 0.05 (H5-treated group) or False Discovery Rate (FDR) < 0.05 (CAZ-H5-treated group) were identified as significantly differentially expressed genes.

Functional annotation was performed using eggNOG-mapper v2 (http://eggnog-mapper.embl.de) for Clusters of Orthologous Groups (COG), Kyoto Encyclopedia of Genes and Genomes (KEGG), and Gene Ontology (GO) annotation. Antibiotic resistance genes (ARGs) were annotated using RGI (https://card.mcmaster.ca/analyze/rgi). GO and KEGG pathway enrichment was performed using the enricher function in clusterProfiler (65).

### Statistical analysis

Analyses were performed with Origin (version 2018) or R (version 4.1) (66). A Shapiro-Wilk normality test was conducted using the shapiro.test function in R. Significance analysis of non-normal data of cell size and plaque size was determined by a Mann-Whitney U test, and statistical comparisons for other normally distributed data were performed on repeated measures by one-way analysis of variance (ANOVA) test. Multiple comparisons were performed using the Tukey HSD test. Statistical analysis of transcriptome data enrichment was performed using Fisher’s exact test in scipy.stats (67), and *P*-values were adjusted using the Benjamini-Hochberg procedure (68). For dimensionality reduction, uniform manifold approximation and projection (UMAP) was conducted by the “umap” package using a naive method in the R (version 4.2.0) environment. Attributed to the theoretical framework of Riemannian geometry and algebraic topology, UMAP tends to strike a balance between emphasizing local versus global structure. After pre-processing, the normalized intensity of all the single-cell Raman spectral data was used as the input matrix, and UMAP was constructed based on the “naïve” method.

## RESULTS

### Identification and characterization of a hvKP phage

A lytic phage H5 was isolated from hospital wastewater using a typical O1:K2 hvKP strain ATCC 43816 as the host. H5 formed large, clear, round plaques approximately 1.5 mm in diameter on the bacterial lawn. The lytic spectrum tests showed that H5 specifically targeted *K. pneumoniae* K2 isolates (Table S1). The optimal MOI of H5 was 0.00001, and progeny phages were produced at 2.70 ± 0.12 × 10^9^ PFU/mL (Fig. S1A). H5 particles were almost completely adsorbed onto cells within 5 min after infection (Fig. S1B). The latent and burst period for H5 was approximately 10 and 15 min, respectively (Fig. S1C). The burst size was approximately 28 phage progeny per infected cell. Lysis kinetics showed that H5 effectively killed the host in a short period (90 min), even at the lowest dose (MOI = 0.0000001) (Fig. S1D and E).

H5 showed limited changes in titer after 3 months of storage at 4°C (Fig. S1F). More than 60% of the phages remained active for 60 min at a wide range of temperatures from 37°C to 50°C, while activity decreased dramatically when the temperature exceeded 60°C (Fig. S1G). H5 was stable (> 86%) in the pH range of 6 to 10 (Fig. S1H). These data suggest that H5 has excellent potential for clinical applications.

### Genomic characterization of H5

The genome of H5 was sequenced to understand its genetic characterization. The H5 genome was 39,296 bp in length with 53.1% G+C content and 49 putative open reading frames (ORFs) (≥ 30 aa) (Fig. S2A; Table S2). No ORFs were associated with phage lysogeny, virulence, antimicrobial resistance, and integrases, further supporting the therapeutic potentials of H5. BLASTn against GenBank revealed that the top 32 hits of H5 (≥ 90% coverage) were all *Klebsiella* phages. H5 exhibited the highest similarity to *Klebsiella* phage KpV763 (accession number: NC_047771; 95% coverage; 96.2% nucleotide identity) and *Klebsiella* virus KP32 (accession number: NC_047968; 97% coverage; 95.7% nucleotide identity), which belongs to the genus *Przondovirus* within the subfamily *Studiervirinae* of the family *Autographiviridae* (a former member of the family *Podoviridae*) (Fig. S2B). H5 was therefore designated as a species of the genus *Przondovirus* according to the species delimitation criterion for bacterial and archaeal viruses (≥95% overall DNA sequence homology).

### Physiological and structural characteristics of phage H5

**Fig 1:**
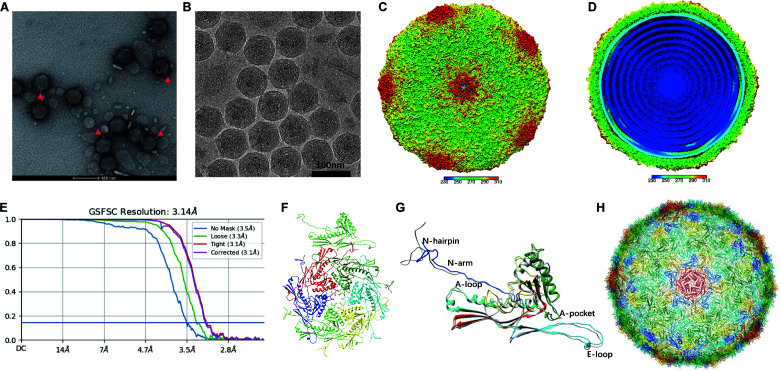
Three-dimensional structural analysis of H5 phage by cryo-EM. Negative staining TEM (**A**) and cryo-EM images (**B**) of the H5 phage. (**C)** Fivefold surface view of corresponding 3D reconstructions. The surfaces are colored by radius. The color key indicates the scale (in angstroms) of the radial color scheme. (**D)** A cut-away view reveals the internal density of the inside content of the H5 phage. (**E)** The Fourier shell correlation curves for gold standard reconstructions of the full reconstructed phage. (**F)** A model of the asymmetric unit of H5 phage. (**G)** The atomic model a of the H5 phage capsid gp10 protein (rainbow) with T7 phage gp10 (gray) superimposed. (**H)** Atomic model of the entire H5 phage capsid in the view as shown in (**B**).

H5 was further characterized using TEM, and showed that it has an icosahedral capsid of approximately 65 nm in diameter with a short tail (10 nm), which is common to members of the *Podoviridae* family (Fig. 1A). We then purified H5 and performed the cryo-EM analysis to dissect its structure (Fig. 1B through D). The 3.14-angstrom resolution density allowed us to build an atomic model (Fig. 1E through H). Comparative analysis of the H5 model and the T7 phage revealed that the H5 gp10 adopted a similar conformation with the mature T7 phage gp10 with an RMSD, 1.186, between the pruned atom pairs. Greater structural variability between H5 and T7 was seen in A-loop, E-loop, A-pocket, and N-hairpin due to aa insertions (A-loop, A pocket) or aa deletions (N-hairpin, E-loop) (Fig. 1G). These differences do not change the configuration of the capsid architecture and do not correlate with the difference in infectivity and specificity between the T7 phage and H5 phage. The variance of infectivity and specificity may have resulted from the portal component of the phage tail.

### Synergistic effect of phage H5 and antibiotics is dependent on antibiotic class and concentration

**Fig 2:**
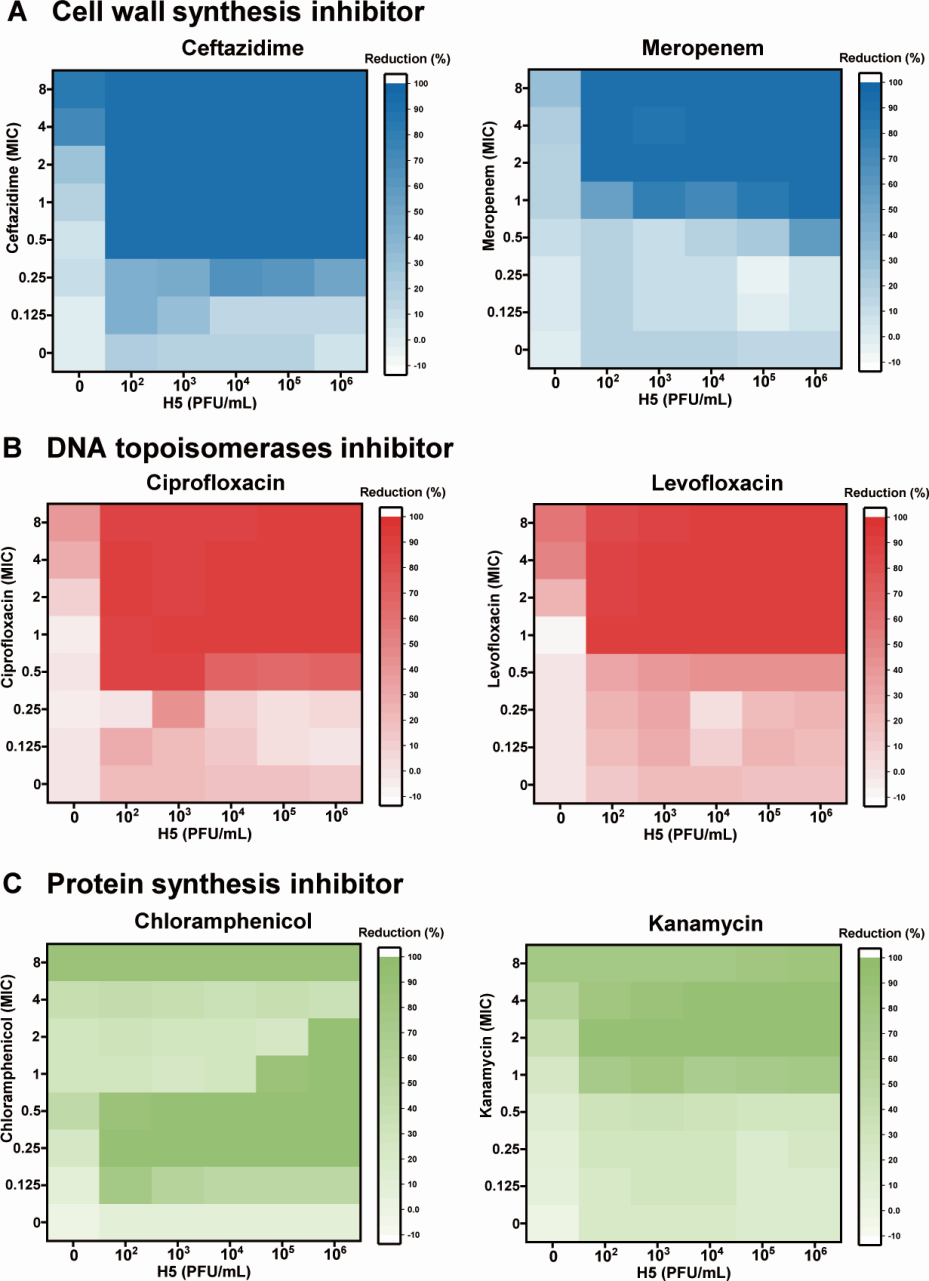
Effect of antibiotic class on PAS. Bacteria in the log phase were inoculated into a 96-well plate coated with H5 and antibiotics, and the OD_600_ was measured every 10 min for a total of 24 h with shaking. The synergistic effect of H5 was estimated with (**A**) ceftazidime and meropenem; (**B**) ciprofloxacin and levofloxacin; and (**C**) chloramphenicol and kanamycin. Synograms (t = 24 h) represent the mean percentage reduction of each treatment from three biological replicates: reduction (%) = [(OD_growth control_ − OD_treatment_)/OD_growth control_] × 100.

We then evaluated the PAS of H5 with different classes of antibiotics. Three types of synogram were obtained from tested phage-antibiotic combinations: (i) the combination of H5 with cell wall synthesis inhibitors or DNA topoisomerase inhibitors (Fig. 2A and B) had a significant synergistic effect (FIC ≤ 0.5); (ii) the combination with polymyxin B, trimethoprim, or rifampicin (Fig. S3A through C) produced a synogram dominated by antibiotic killing and no significant synergistic effect was observed (FIC > 0.5); (iii) the combination with kanamycin or chloramphenicol (Fig. 2C) displayed an antibiotic concentration-limited synergistic effect (FIC ≤ 0.5), i.e., a significant synergistic effect can be observed over a certain concentration range of the antibiotic.

**Fig 3:**
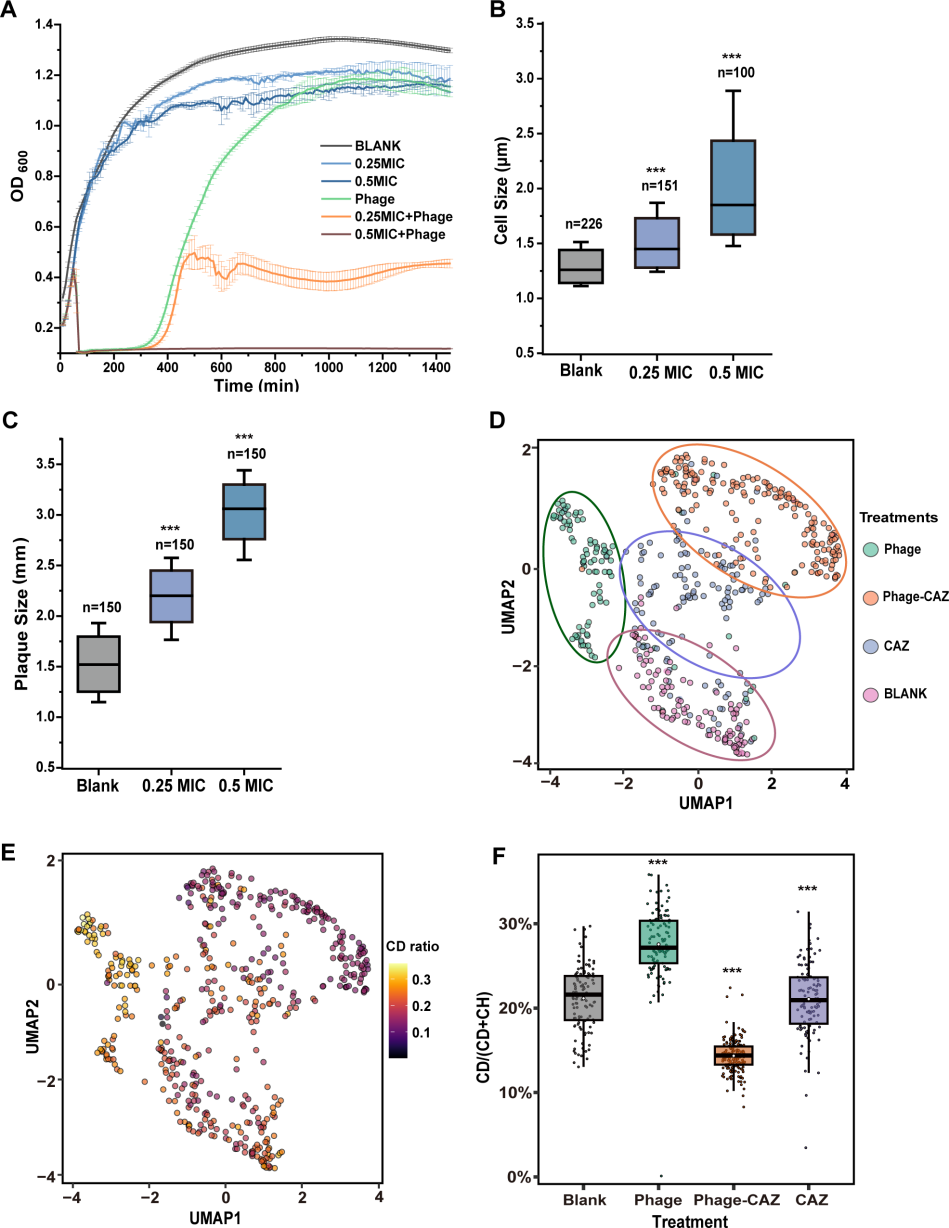
Physiological changes of bacteria in response to the phage H5 and CAZ synergy. (**A)** Effect of combinatorial treatment on bacterial regrowth. Effect of CAZ concentrations on cell size (**B**) and plaque size (**C**). The number of cells or plagues counted for each concentration is indicated. UMAP representation of phenotypic responses of individual cells to different CAZ and phage treatments based on the whole Raman fingerprint region (**D**) and C–D region (**E**). **(F)** Single-cell metabolic activity under different treatments was revealed by single-cell Raman with D_2_O labeling. Each point is a measurement of a single cell, and box plots show the quartiles of each evolved population. Statistical analysis was performed using Mann-Whitney U tests or one-way ANOVA followed by Tukey HSD tests. ***, *P* < 0.001.

The synergistic effect of H5 and CAZ was mainly driven by the concentration of antibiotics (Fig. 3A). Approximately 66% reduction in the OD_600_ value was detected (from mean 1.30 ± 0.01 to 0.44 ± 0.08) when the final concentration of CAZ reached 0.25 MIC. Bacterial growth was inhibited with undetectable colonies by plating the 24-h culture with 0.5 MIC CAZ (< 8.7 ± 0.4 × 10^9^ CFU/mL) (Fig. S4A). The synergistic effect was detected at 90 min after infection (Fig. S4B), suggesting that the synergistic effect of the H5-CAZ combination can achieve rapid elimination of bacteria.

To further understand the robustness of synergistic effect displayed by the combination of cell wall synthesis inhibitors with phages, we tested the combination of CAZ with a *Myoviridae* phage B1 against a carbapenem-resistant hypervirulent *K. pneumoniae* (CR-hvKP) ST11-O2v1:KL64 clinical strain (KPn47434), representing the most prevalent CRKP/CR-hvKP clone in China (20). B1 differed from H5 in both morphology and genetics (159,349 bp in length with 45.99% G+C content and 213 predicated ORFs) (Fig. S4C). A synergy was also observed for the B1-CAZ combination (FIC ≤ 0.5) (Fig. S4D), suggesting the robustness of the synergistic effect achieved by combinations of cell wall synthesis inhibitors with various *Klebsiella* phages.

### Synergistic interaction between H5 and CAZ is due to cell elongation and enhanced phage proliferation

To understand the mechanism underlying the PAS observed, we examined the cell morphology for cultures before and after being treated with 0.25 or 0.5 MIC CAZ for 6 h. Intact short rod-shaped cells were observed under antibiotic-free conditions, with an average cell size of 1.31 ± 0.25 µm (Fig. 3B; Fig. S5A). Incubation with 0.25 MIC CAZ resulted in cell elongation (mean 1.56 ± 0.39 µm), and cells further elongated (mean 2.18 ± 0.88 µm) with increasing concentration to 0.5 MIC (Fig. 3B; Fig. S5A). The cell membrane was intact, but the boundary became obscure, and more intracellular soluble substances were detected, suggesting that CAZ caused damage to the cell membrane. Cell elongation led to filamentation (Fig. S5B) and significantly promoted phage adsorption with an increase of mean 23.87% ± 5.01% (0.25 MIC) and 45.90% ± 2.93% (0.5 MIC) (Fig. S5C). These results suggest that the presence of CAZ can promote phage adsorption through cell elongation.

CAZ had no effect on the lysis kinetics of H5 (Fig. S5D), indicating that PAS was not caused by altering the phage life cycle. However, the burst size of single cells was significantly improved (mean 27.51 ± 1.02 to 47.51 ± 1.97 PFU/cell; *P* < 0.01; one-way ANOVA test) as the CAZ concentration increased (Fig. S5E). Compared to that of the phage-treated group, the transcription level of the phage genes encoding the internal virion protein, major capsid protein, tail assembly protein, DNA polymerase, and endolysin was significantly upregulated in the CAZ-H5-treated group (Fig. S5F), suggesting that CAZ promotes the burst size by increasing the proliferation of progeny phage. This is further supported by the significantly larger plaques formed with CAZ than without CAZ (mean 3.00 ± 0.44 mm versus 1.54 ± 0.39 mm; *P* < 0.001; Mann-Whitney U tests) (Fig. 3C; Fig. S5G). These results suggest that enhanced phage progeny proliferation is another factor promoting the PAS.

### PAS suppresses the metabolic activity of bacteria

While augmented phage proliferation may boost therapeutic efficacy, it fails to elucidate the suppression of bacterial regrowth achieved by the H5-CAZ combination (Fig. 3A), which is a frequent occurrence in phage therapy. We noted that small and transparent colonies were observed in the H5-CAZ dual treatment group but not in the single treatment (Fig. S6A), suggesting that the phage-antibiotic combination imposed a lower metabolic activity than either of the single pressures. To further clarify this effect, a single-cell Raman platform (42) combined with D_2_O labeling was used to trace the *in situ* physiological responses of bacteria to phage and/or CAZ at the single-cell level. Analysis of the entire spectral region representing the whole cellular state using the advanced UMAP classified all the individual cells into four groups corresponding to the treatment conditions (Fig. 3D), indicating that a specific physiological response was induced by each pressure. The corresponding single-cell CD ratios showed that phage-treated cells had the highest metabolic activity even above the blank group, while the phage-CAZ-treated cells showed the lowest activity (Fig. 3E and F), suggesting that bacterial metabolic activity was upregulated by phage treatment, but was suppressed by phage-antibiotic combination.

### PAS changes the bacterial evolutionary trajectory

**Fig 4:**
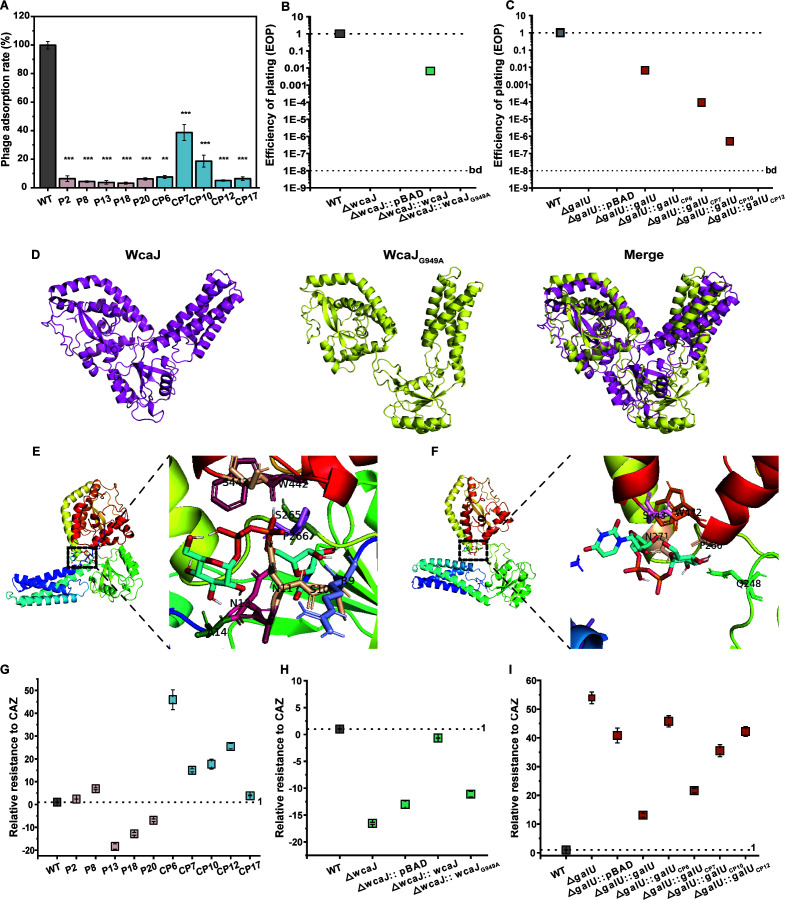
Effect of PAS on a bacterial evolutionary trajectory. (**A)** The phage adsorption rate and EOP of WT and mutants. (**B)** Phage resistance of 43816Δ*wcaJ* and different complements indicated by EOP. (**C)** Phage resistance of 43816Δ*galU* and different complements indicated by EOP. (**D)** The effect of point mutation G949A on the 3D structure of WcaJ. Predicted binding conformation of UDP-D-glucose at the substrate-binding site of the modeled WcaJ (**E**) and WcaJ_G949A_ (**F**) structure. The side chains of residues connected with UDP-D-glucose with hydrogen bonds are indicated in the enlarged views. (**G)** The susceptibility of WT and mutants to CAZ. (**H)** The susceptibility of 43816Δ*wcaJ* and different complements to CAZ. (**I)** The susceptibility of 43816Δ*galU* and different complements to CAZ. Susceptibility was defined as the ratio of the number of viable cells in the mutants compared to WT strains under CAZ (0.25 mg/L) treatment. A phage that produces an equal number of plaques on WT has an EOP of 1.0 (dotted line). A “bd” at the lower, dashed line indicates that the EOP was below the limit of detection (~10^−8^). Statistical analysis was performed using one-way ANOVA tests. ^**^, *P* < 0.01 and ***, *P* < 0.001.

Given the different metabolic activities observed in cells treated with phages and phage-CAZ, we hypothesized that distinct genetic alternations may have been involved in response to varying pressures. To test this hypothesis, five mutants were randomly selected after 24 h of treatment with phage (P2, P8, P13, P18, and P20) or phage-CAZ (0.25 MIC) combinations (CP6, CP7, CP10, CP12, and CP17). All phage-treated mutants were resistant to a second H5 challenge (EOP < 10^−8^), which was largely due to reduced capsule production (mean 28.45% ± 1.09% ~ 53.32% ± 1.10%) and blocked phage adsorption (mean > 95.27% ± 1.45%) in the mutants (Fig. 4A). While the phage-CAZ-treated mutants exhibited variable phage resistance, with three of them (CP6, CP12, and CP17) showing undetectable plaquing ability (EOP < 10^−8^), and the other two (CP7 and CP10) showing ~10^−3^ to 10^−5^ plaquing ability compared to WT. The various phage resistance was highly correlated with the extent of blocked phage adsorption, with CP7 and CP10 blocking 61.33% ± 5.55% and 81.33% ± 4.18% of adsorption, respectively, and the remaining three blocking more than 92.5% ± 0.94% (Fig. 4A). Reduced capsule production (33.24% ± 2.72% ~ 52.52% ± 0.64%) was also detected in these mutants (Fig. S6B). The 10 mutants consistently showed attenuated virulence compared to WT due to reduced capsule production (Fig. S6D and E).

The 10 mutants were sequenced to further illustrate the underlying mechanism mediating the different phenotypes. A total of seven missense mutations were detected, of which five were indels, and the remains were SNPs (Table 1). All phage-treated mutants exclusively shared a missense mutation (G949A; Gly317Arg) in the *wcaJ* gene (encoding undecaprenyl-phosphate glucose phosphotransferase), and an 8,354 bp insertion and a non-synonymous SNP (Leu235Met) in *dmsC* gene was additionally detected in P8 and P18, respectively. To demonstrate whether the missense mutation of *wcaJ* mediated phage resistance, we successfully engineered a null mutant (43816Δ*wcaJ*). Compared with WT, deletion of *wcaJ* reduced capsular production (mean 41.08% ± 2.06%) (Fig. S6B), blocked phage adsorption (mean 92% ± 2.49%) (Fig. S6C), and caused phage resistance with undetectable plaquing ability (EOP < 10^−8^) (Fig. 4B). These phenotypes of 43816Δ*wcaJ* were consistent with the five phage-treated mutants, and could be rescued by complementation with *wcaJ* but not with *wcaJ*_G949A_ (Fig. 4B), demonstrating that the missense mutation of *wcaJ* confers phage resistance.

**TABLE 1:** Mutation site analysis of variants from phage treatment alone and phage-CAZ treatments

nt Position	Mutation	Gene	Function	Mutants									
				P2	P8	P13	P18	P20	CP6	CP7	CP10	CP12	CP17
253218	Outframe deletion GCACGTGACT	*galU*	UTP-glucose-1-phosphate uridylyltransferase								**+**		
253325	Inframe deletion GTTATTCTGCCAGAC								**+**				
253549	Outframe deletion AACCT											**+**	
253774	Outframe insertion C									**+**			
1410275_1418628	Insertion	Nine genes			**+**								
1565093	SNP A > C Leu235Met	*dmsC*	Dimethyl sulfoxide reductase anchor subunit				**+**						
5188437	SNP G > A Gly317Arg	*wcaJ*	Undecaprenyl-phosphate glucose phosphotransferase	**+**	**+**	**+**	**+**	**+**					

To further understand how the G949A mutation affects the function of WcaJ, alignments were performed on 5,346 WcaJ sequences retrieved from GenBank. Site analysis predicted that G949A is located in the cytoplasm and this mutation alters a highly conserved structural residue (Fig. S7), suggesting that it may be critical for the catalytic function of undecaprenyl-phosphate glucose phosphotransferase (69). PyMol and Autodock were used to compare the 3D structure of WcaJ and WcaJ_G949A_ and to calculate the possible binding sites between the proteins and their substrate UDP-D-glucose. WcaJ and WcaJ_G949A_ are significantly different in the protein structural architecture with an RMSD value of 15.034 Å (Fig. 4D). Flexible ligand docking predicted that the binding sites involved in WcaJ-substrate and WcaJ_G949A_-substrate interactions are different (Fig. 4E and F). These analyses suggest that G949A leads to a dramatic change in the structure of WcaJ, resulting in alterations in substrate binding.

**Fig 5:**
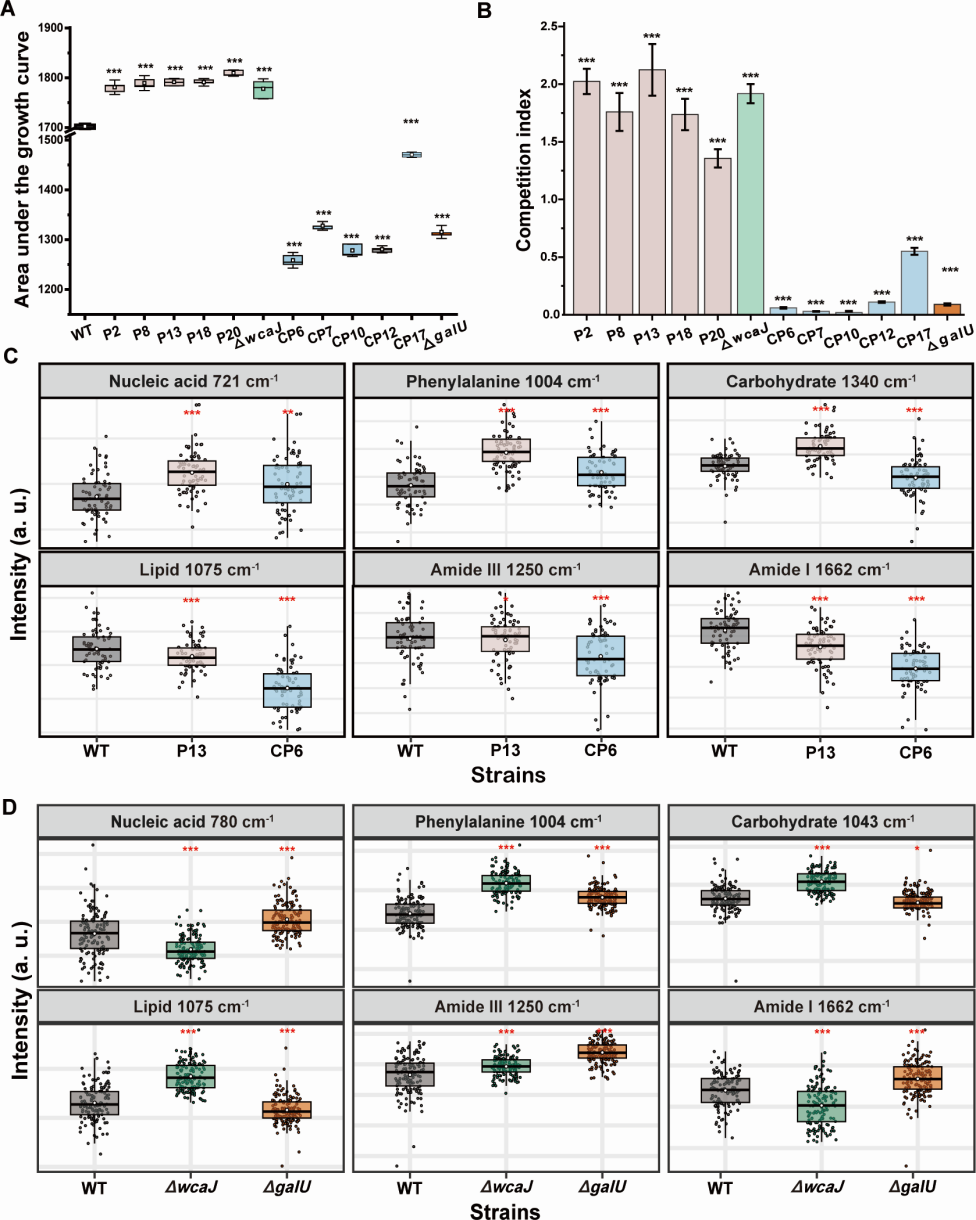
Impact of PAS on fitness cost and biological processes of *K. pneumonia* ATCC 43816. The area under the growth curves (**A**) and competitiveness (**B**) of WT (ATCC 438186) and two mutants (**P13 and CP6**) and the competition index was defined as the ratio of the number of viable cells in the mutants to the WT in mixed culture. The relative intensity of major biomolecules in mutants (**C**) and gene knockout strains (43816Δ*wcaJ* and 43816Δ*galU*) (**D**). Each point is a measurement of a single cell, and box plots show the quartiles of each evolved population. Statistical analysis was performed using one-way ANOVA tests. ^*^, *P* < 0.05; ^**^, *P* < 0.01; and ^***^, *P* < 0.001.

Various indels were detected in the *galU* gene (encoding UTP-glucose-1-phosphate uridylyltransferase) in four of the five phage-CAZ treatment mutants (CP6, CP7, CP10, and CP12), and no mutations were detected in CP17. To demonstrate whether *galU* mutations mediated phage resistance, a *galU* knockout (43816∆*galU*) was successfully constructed. Compared with WT, deletion of *galU* caused an approximately twofold reduction of capsular production (Fig. S6B), blocked phage adsorption (mean 94.67% ± 4.40%) (Fig. S6C), and caused phage resistance with an undetectable plaquing ability (EOP < 10^−8^) (Fig. 4C). The phage resistance of 43816Δ*galU* was abolished by complementation with *galU*, and varied by complementation of the different *galU* alleles detected in the four mutants (Fig. 4C). This is consistent with the phage resistance observed for the four mutants (Fig. 4A), suggesting that various mutations of *galU* confer different levels of phage resistance. Since no mutations were detected in CP17, we suppose that phage resistance may be correlated with the altered transcription levels of receptor-binding proteins. Indeed, the transcription level of *ompK36* decreased approximately fourfold in CP17 compared to WT (Fig. S8), suggesting a different adaptation strategy.

To determine whether mutations that occurred in *wcaJ* and *galU* were randomly detected or not, 10 additional phage-treated and 10 phage-CAZ-treated mutants were randomly chosen for two biological replicates. Sequencing results showed that *wcaJ* (G949A) was detected in all phage-treated mutants, and mutations in *galU* were detected in three phage-CAZ-treated mutants.

### *wcaJ* mutations are selected by phage and *galU* mutations are induced by phage-CAZ combination

To understand how the evolutionary trajectory was changed by PAS, a fluctuation test was performed to determine whether the *wcaJ* and *galU* mutations detected here were the result of selective pressure or induced mutations. The number of resistant colonies between samples from different populations (Group A) and samples from the same population (Group B) was significantly different under selection either by phage or by phage-CAZ combination (Fig. S9A through C), indicating that resistance mutants were pre-existing in the population. Analyzing the genomes of 20 randomly selected mutants under phage selection from Group A detected 22 missense mutations (18 were SNPs, and four were deletions) in seven genes, and the function of the genes with mutations is associated with the cell surface, including five CPS-related genes, one LPS-related gene, and one outer membrane-related gene (Table S3). Of note, 10 mutations were found in the *wcaJ* gene, and none in *galU*. Additionally, no mutations were detected in *galU* by sequencing 20 randomly selected mutants under phage-CAZ treatment. These results demonstrate that the *wcaJ* mutations were pre-existing and selected for by phages, whereas the *galU* mutations were induced by phage-CAZ pressure.

To estimate whether pre-existing *wcaJ* mutations are commonly involved in KP against phages, we additionally performed a fluctuation test using KPn47434 and phage B1. Two of 20 mutants randomly selected from Group A carried a frameshift-mutated *wcaJ* gene, suggesting that pre-existing *wcaJ* mutations may be an important strategy employed by KP against phages. However, *galU* mutations were not detected in these mutants, indicating that different adaptive mechanisms are deployed in the different strains.

To find out why mutations in *wcaJ*, but not other capsular synthesis genes, were exclusively selected for by phages, we chose an *etk* gene (encoding a tyrosine-protein kinase) required for the capsule formation (70), as an example to investigate the question. The 43816Δ*etk* mutant was resistant to H5 (EOP < 10^−8^) (Fig. S9D) by blocking phage adsorption (98.17% ± 2.06%) (Fig. S9E). However, 43816Δ*etk* showed a significantly lower area under the growth curve (AUC) (71) than WT and 43816Δ*wcaJ* (*P* < 0.001; one-way ANOVA test) (Fig. S9F), suggesting that *wcaJ* mutations confer evolutionary advantage compared to *etk*.

### Collateral sensitivity to CAZ mediated by *wcaJ* mutations promotes PAS

Given that the PAS of H5 and CAZ is largely determined by CAZ concentration (Fig. 2A), we assume that CAZ could be a driving force diversifying the evolutionary trajectory of phage- and phage-CAZ-treated populations. To test this hypothesis, we measured the CAZ susceptibility of the 10 mutants. The MIC of CAZ for the phage-treated mutants was identical to the WT (0.5 mg/L), while P13, P18, and P20 showed an approximately 7- to 18-fold reduction in survival rate after treatment with 0.25 mg/L CAZ compared to the WT, suggesting that they became collateral-sensitive to CAZ (Fig. 4G). In contrast, cross-resistance to CAZ was observed for P2 and P8, with an approximately two to sevenfold increase in survival compared to the WT (Fig. 4G). All phage-CAZ-treated mutants also showed the same MIC as the WT, but became cross-resistant to 0.25 mg/L CAZ, with an approximately 4- to 45-fold increase in survival rate compared to the WT (Fig. 4G).

We next determined whether the detected mutations in *wcaJ* and *galU* confer collateral sensitivity and cross-resistance to CAZ, respectively. 43816Δ*wcaJ* or 43816Δ*wcaJ:wcaJ*_G949A_ showed increased CAZ (0.25 mg/L) sensitivity compared to the WT, with an approximately 11-fold decrease of viable cells (Fig. 4H). The increased CAZ sensitivity was restored by the complementation of *wcaJ* but not by *wcaJ*_G949A_ (Fig. 4H), suggesting that the *wcaJ* mutation indeed mediates collateral sensitivity to CAZ. In contrast, 43816Δ*galU* showed enhanced CAZ resistance compared to the WT, with more than a 53-fold increase of viable cells, which was rescued by the complementation of *galU* but not by the four variants detected (Fig. 4I). This confirms that *galU* mutations confer cross-resistance to CAZ. We further found that the *ompK36* transcription level was decreased in the *galU* null mutant and all phage-CAZ-treated mutants (Fig. S8), suggesting that cross-resistance to CAZ mediated by *galU* mutations could be due to decreased membrane permeability.

### Higher fitness cost caused by GalU mutations further promotes PAS

Despite the phage-CAZ-treated mutants obtaining enhanced resistance to CAZ and phage, it is puzzling that their growth appeared to be suppressed, failing to match the recovery of phage-treated mutants (Fig. 3A). Indeed, the phage-treated mutants exhibited a significantly superior growth potential, as indicated by AUC, whereas the phage-CAZ-treated mutants had an inferior growth rate (Fig. 5A), suggesting that the phage-treated mutants are generally fitter than the phage-CAZ-treated mutants. To further demonstrate the fitness cost of the mutants, we performed competition assays between WT and each mutant. WT was outcompeted by each of the phage-treated mutants in independent competition pairs after 3-day passage, and the mutants showed significantly higher fitness than WT as indicated by CI (Fig. 5B). In contrast, each phage-CAZ-treated mutant was outcompeted by WT after 3-day passage, and the fitness of mutants was significantly lower than that of WT (Fig. 5B). These results suggest that phage-treated mutants showed an evolutionary trade-up between phage resistance and fitness, whereas phage-CAZ-treated mutants displayed a trade-off between the two.

To gain further insight into whether the observed fitness contrasts were caused by mutations in *wcaJ* and *galU*, we performed competition assays between WT and 43816Δ*wcaJ or* 43816Δ*galU*. The WT was outcompeted by 43816Δ*wcaJ* while 43816Δ*galU* was outcompeted by the WT after a 3-day passage (Fig. 5B). This aligns with the competition outcomes between WT and mutants, demonstrating that mutations in *wcaJ* and *galU* confer contrasts in fitness. In accordance, 43816Δ*wcaJ* showed significantly higher AUC than the WT, while the AUC of 43816Δ*galU* was significantly lower than the WT (*P* < 0.001; one-way ANOVA test) (Fig. 5A). Together, these results demonstrate that mutations in *galU* confer a higher fitness cost, where are *wcaJ* mutations increase the fitness of the population.

### Fitness cost is associated with suppressed carbohydrate metabolism caused by GalU mutations

We next tried to identify biological processes correlated with the fitness alterations using Raman spectroscopy and transcriptome sequencing on P13 (with *wcaJ* mutation) and CP6 (with *galU* mutation). P13 and CP6 showed distinct Raman bands mainly at 721 cm^−1^ (nucleic acid), 1,004 cm^−1^ (phenylalanine), 1,075 cm^−1^ (Lipid), 1,250 cm^−1^ (amide III), 1,340 cm^−1^ (carbohydrate) and 1,662 cm^−1^ (amide I). Except for that of 721 cm^−1^ and 1,004 cm^−1^, the intensity of the other bands was significantly lower in CP6 than in WT and P13 (*P* < 0.05; one-way ANOVA followed by Tukey HSD tests) (Fig. 5C), suggesting that the metabolism of proteins, phospholipid, and carbohydrate was suppressed in CP6 (42, 72).

RNA-seq analysis identified 139 differentially expressed genes (DEGs) (*P* < 0.05) in P13, and 1,019 DEGs (FDR < 0.05) in CP6 (Fig. S10A), indicating that a more complex response was triggered by the dual pressure. The up-regulated DEGs of P13 were enriched in eight biological processes, mainly involved in protein transport and carbohydrate metabolism (Fig. S10B), and down-regulated DEGs in CP6 are mainly categorized into carbohydrate transport and metabolism, and energy production and conversion (Fig. S10C). The results are consistent with that of Raman spectroscopy. KEGG analysis further showed that the phosphotransferase system (PTS) and ascorbate and aldarate metabolism pathway was up-regulated in P13 (Table S4), but down-regulated in CP6 (Table S5), suggesting potential targets for suppressing phage resistance.

To determine whether the altered metabolic activity was caused by the mutations of *wcaJ* and *galU*, Raman spectroscopic assays were performed for the two null mutants. Suppressed and upregulated carbohydrate metabolism were observed in ∆*galU* and ∆*wcaJ* mutant, respectively (*P* < 0.001; one-way ANOVA followed by Tukey HSD tests) (Fig. 5D). This was confirmed by quantitative PCR (q-PCR) results for selected genes involved in carbon metabolisms (*sdhC*, *ppsA*, *VK055_0273*, and *VK055_0372*) (Fig. S11A), specifically the PTS (*manY* and *manZ*) and ascorbate and aldarate metabolism pathway (*sgaB*, *garD*, and *garL*) (Fig. S11B), suggesting that altered carbohydrate metabolism was caused by the mutations of *wcaJ* and *galU*.

### Compensation mechanism mediates the switch from trade-off to trade-up between phage and CAZ resistance

The *wcaJ*_G949A_ mutation caused a trade-off between phage and CAZ resistance. However, two of the five phage-treated mutants (P2 and P8) showed an evolutionary trade-up, indicating that a compensation mechanism may have been triggered in the mutants. To test this hypothesis, we searched for unique mutations in P2 and P8. An 8,354 bp duplication encoding nine ORFs was found in P8 and no additional mutations were detected in P2 (Table S6). Enhanced transcription levels (2 – 12-fold) for the nine ORFs were detected in P8 (Fig. S12A), suggesting that the duplication was functional. Since only four of the nine ORFs (VK055_1417, VK055_1419, VK055_1420, and VK055_1425) were annotated with known functions (Table S6), we therefore targeted the four ORFs to test our hypothesis. Overexpression of each of the four ORFs in WT or 43816∆*wcaJ* increased more than twofold resistance to CAZ (Fig. S12B), suggesting that the duplication in P8 contributes to the enhanced resistance to CAZ.

We further measured epistasis to evaluate the interaction between *wcaJ*_G949A_ and the duplication. Epistasis occurs when different genes make a non-additive contribution to phenotypes (73). The observed fitness of each ORF overexpression ranged from −1.52 ± 0.35 to 2.55 ± 0.20, resulting in a positive mean epistasis (0.23) (Fig. S12C), suggesting that the duplication compensates for the reduced CAZ resistance caused by *wcaJ*_G949A_ in P8.

## DISCUSSION

The combined use of phages and antibiotics has been recognized as a superior strategy for controlling bacterial pathogens, especially in suppressing the emergence of phage and/or antibiotic resistance in bacteria. However, the mechanistic elucidation of this synergistic interaction is very limited. In this study, we tried to understand how PAS suppresses the emergence of resistance by using an isotope-labeled single-cell Raman spectroscopy approach together with multi-omic techniques to track the physiological evolutionary trajectory of pressure *in situ*, and genetic alterations associated with evolutionary drivers were further determined, demonstrating a novel mechanism of resistance suppression by a phage-antibiotic combination in *K. pneumoniae*.

PAS is highly dependent on the type of antibiotics, i.e., the primary target of the antibiotics (32). The ubiquitous synergistic effect of phages in combination with CAZ found in this and other studies suggests that the antibiotics with less impact on the cellular processes required for phage replication will be more advantageous in combination with phages (74–77). Several mechanisms of PAS have been proposed (19), including cell elongation/filamentation by antibiotics resulting in accelerated phage amplification and enhanced burst size; reducing the emergence of phage and/or antibiotic-resistant mutants; resensitizing bacteria to antibiotics due to the presence of the phage. These mechanisms have consistently been demonstrated in our study. However, we are still far from understanding how these phenotypic changes caused by PAS occur. We, therefore, used a Raman platform to track the physiological stress responses of bacteria at the single-cell level and found that phage-CAZ treatment suppressed cellular metabolic activity, whereas sole phage treatment upregulated metabolic activity (Fig. 3F). It is known that the altered metabolic activity can affect growth rates (78), competition (79), and virulence (80). Indeed, compared to phage-treated mutants and WT, phage-CAZ-treated mutants showed inferior growth rates with decreased competition in our study (Fig. 5A and B). It has been suggested that metabolism-dependent antibiotics (81), e.g., CAZ, are prone to induce metabolism-associated mutations, resulting in altered metabolic activity. This means that the addition of CAZ drives the evolutionary trajectories of bacteria under different pressures, which can partially explain why the synergistic effect is highly dependent on the CAZ concentration (Fig. 3A).

We further pinpoint that the differences in the metabolic activity detected between phage- and phage-CAZ-treated mutants were highly associated with different genetic alterations in the two populations. A point mutation was found in *wcaJ* of phage-treated mutants, resulting in an evolutionary trade-up between phage resistance and fitness, but a trade-off between collateral sensitivity to CAZ (Fig. 4G and H; Fig. 5B). *wcaJ* encodes the first glycosyltransferase of the capsule biosynthetic pathway and is responsible for capsule biosynthesis (82). Therefore, *wcaJ* mutations cause reduced capsule production, leading to phage resistance through blocked phage adsorption (83). Mutations in *wcaJ* have been reported in several studies as a common strategy of KP against phage resistance (37, 83), further supporting its role as an evolutionary advantage in coping with phage selection. Such an evolutionary advantage is strongly associated with the upregulated carbohydrate metabolism activity conferred by *wcaJ* mutations as determined here (Fig. 5D), thus promoting superior growth and increasing fitness. Of note, we demonstrated that *wcaJ* mutations were pre-existing in the WT population. This explains well why these mutations were frequently selected by phages in numerous studies. Taken together, these data suggest that certain pre-existing mutations with evolutionary advantage facilitate KP to cope with phage infection.

Intriguingly, the *wcaJ* mutations were selected against by phage-CAZ treatment and the induced *galU* mutations were selected for in the population. This could be largely due to the trade-off between phage resistance and collateral sensitivity to CAZ caused by the *wcaJ* mutations (Fig. 4H), and new mutations were forced to be developed to cope with two different selective pressures. Indeed, the *galU* mutations were induced by phage-CAZ pressure, as confirmed by fluctuation tests. *galU* serves as a building block for LPS, capsular polysaccharide, and osmoregulated periplasmic glucans (84), thus mutations in *galU* confer phage resistance and cross-resistance to CAZ through impaired phage adsorption and reduced capsule production, as well as decreased membrane permeability by suppressed *ompK36* transcription level (Fig. S9), respectively. In contrast to *wcaJ* mutations, evolutionary trade-offs were found for phage resistance mediated by *galU* mutations in terms of inferior growth and higher fitness costs (Fig. 5A and B). This could be largely attributed to the suppressed carbohydrate metabolic activity conferred by *galU* mutations, as supported by both RNA-seq and Raman spectroscopy analysis. Additionally, we noted that the combination of phage with high concentrations of CAZ can eliminate the occurrence of *galU* mutations, which could be due to the relatively weak resistance to CAZ with identical MIC to WT. Our findings suggest that PAS is a superior strategy for treating infections caused by strains with pre-existing mutations conferring phage resistance, which is also effective in reducing the risk of recurrent infections caused by antibiotic/phage-resistant bacteria. Additionally, optimizing antibiotic dosing when using PAS in clinical settings is very important, not only for PAS optimization but also to prevent the emergence of resistance.

We also noted that other mechanisms may have been involved in promoting PAS, since the metabolic activities of phage- and phage-CAZ-treated mutants were not fully consistent with those of the *wcaJ* and *galU* null mutants, especially in terms of protein metabolic activity (Fig. 5D). Indeed, RNA-seq analysis of P13 and CP6 identified a number of genes with altered transcript levels (Fig. S10A), and their roles in promoting PAS should be studied further. Of note, P2 showed more resistance to CAZ than P13 and P20, although they carried the same *wcaJ* mutation. We suppose that additional (compensation) mechanisms of CAZ resistance could be developed by P2, such as epigenetic modification or transcriptional alterations, and further studies are needed to clarify it.

Overall, our study reveals a novel mechanism of PAS that phage-CAZ treatment suppresses metabolic activities and alters the evolutionary trajectory of the population, which is linked to the replacement of pre-existing mutations with evolutionary fitness by those causing higher fitness costs.

## Data Availability

WGS data were deposited in GenBank under the BioProject accession number PRJNA994484. RNA-seq data have been deposited in the NCBI GEO database under accession number GSE237490. The complete genome sequence of H5 has been deposited in GenBank under accession number OR296322. Other relevant data are available from the corresponding author upon reasonable request.

## SUPPLEMENTAL MATERIAL

Supplemental figures mbio.01393-24-s0001.docx Figures S1-12.

Table S1 mbio.01393-24-s0002.xlsx Host range of phage H5.

Table S2 mbio.01393-24-s0003.xlsx Predicted ORFs and genes in the H5 genome.

Table S3 mbio.01393-24-s0004.xlsx Mutation site analysis.

Table S4 mbio.01393-24-s0005.xlsx Enriched KEGG pathways of upregulated DEGs in P13 versus WT.

Table S5 mbio.01393-24-s0006.xlsx Enriched KEGG pathways of downregulated DEGs in CP6 versus WT.

Table S6 mbio.01393-24-s0007.xlsx Sequence information inserted into P8 strain.

Table S7 mbio.01393-24-s0008.xlsx Primers.

ASM does not own the copyrights to Supplemental Material that may be linked to, or accessed through, an article. The authors have granted ASM a non-exclusive, world-wide license to publish the Supplemental Material files. Please contact the corresponding author directly for reuse.
