# Supplementary material for: Phage-antibiotic synergy suppresses resistance emergence of Klebsiella pneumoniae by altering the evolutionary fitness
Source: mBio. 2024 Sep 9;15(10):e01393-24. doi: 10.1128/mbio.01393-24 (PMC11481518; doi:10.1128/mbio.01393-24)
Supplement: Supplemental figures — Figures S1-12. [file mbio.01393-24-s0001.docx]

Supplementary Materials for

Phage-antibiotic synergy suppresses resistance emergence of *Klebsiella pneumoniae* by altering the evolutionary fitness

Kunhao Qin^1#^, Xing Shi^1#^, Kai Yang^2#^, Qiuqing Xu^1^, Fuxing Wang^3^, Senxiong Chen^3^, Tingting Xu^1^, Jinquan Liu^1^, Wangrong Wen^4, 5^, Rongchang Chen^1^, Zheng Liu^3*^, Li Cui^2*^, Kai Zhou^1*^

^1^ Shenzhen Institute of Respiratory Diseases, Southern University of Science and Technology, Shenzhen, 518020, China

^2^ Key Laboratory of Urban Environment and Health, Fujian Key Laboratory of Watershed Ecology, Institute of Urban Environment, Chinese Academy of Sciences, Xiamen, 361021, China

^3^ Kobilka Institute of Innovative Drug Discovery, School of Medicine, Chinese University of Hong Kong, Shenzhen, 518172, China

^4^ Clinical Laboratory Centre, The First Affiliated Hospital of Jinan University, Guangzhou, 510630, China

^5^ Clinical Laboratory, The Affiliated Shunde Hospital of Jinan University, Foshan, 528305, China

^*^Corresponding authors

E-mail: [zhouk@mail.sustech.edu.cn](mailto:zhouk@mail.sustech.edu.cn) (Kai Zhou), [lcui@iue.ac.cn](mailto:lcui@iue.ac.cn) (Li Cui), [liuzheng@cuhk.edu.cn](mailto:liuzheng@cuhk.edu.cn) (Zheng Liu)

^#^ These first authors contributed equally to this article.

**This file includes:**

Figure. S1-12

**Other Supplementary Materials for this manuscript include the following:**

Tables S1-7 (separate file)

**Supplementary Figures**

**
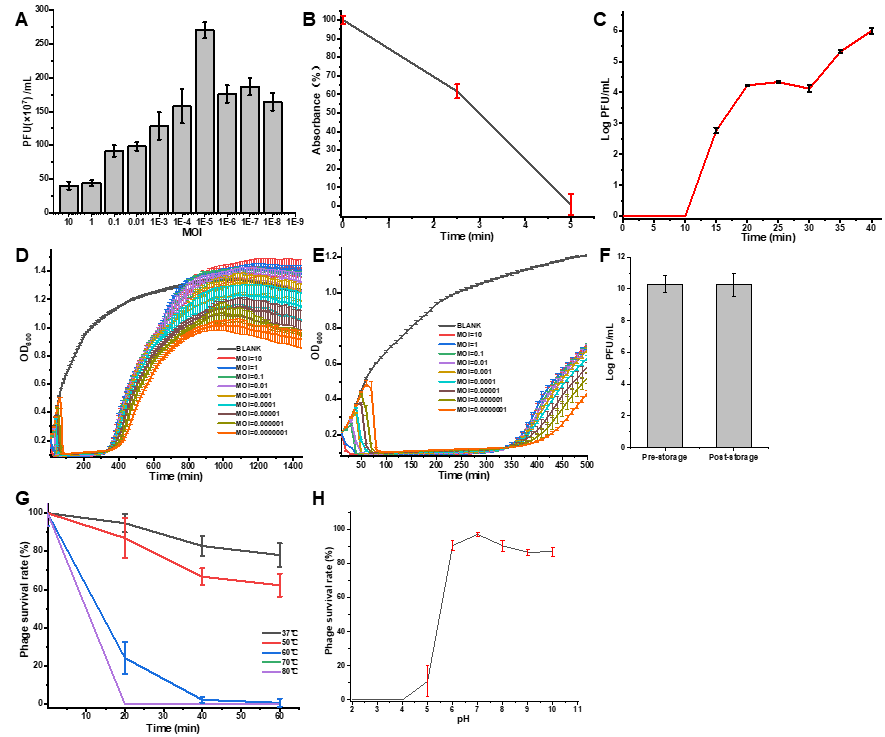
**

**Figure. S1** The characteristic analysis of the novel phage H5. (**A)** Number of progeny phages under different MOI. (**B)** The adsorption curve of H5. (**C)** The one-step growth curve of phage H5. (**D)** Cell lysis assay of H5 under different MOI *in vitro* for 24 h. (**E)** Development of phage resistance in wild-type bacteria under different MOI *in vitro*. Phage activity after 3 months at 4 ℃ (**F**), thermal stability (**G**), and pH stability (**H**) of H5.


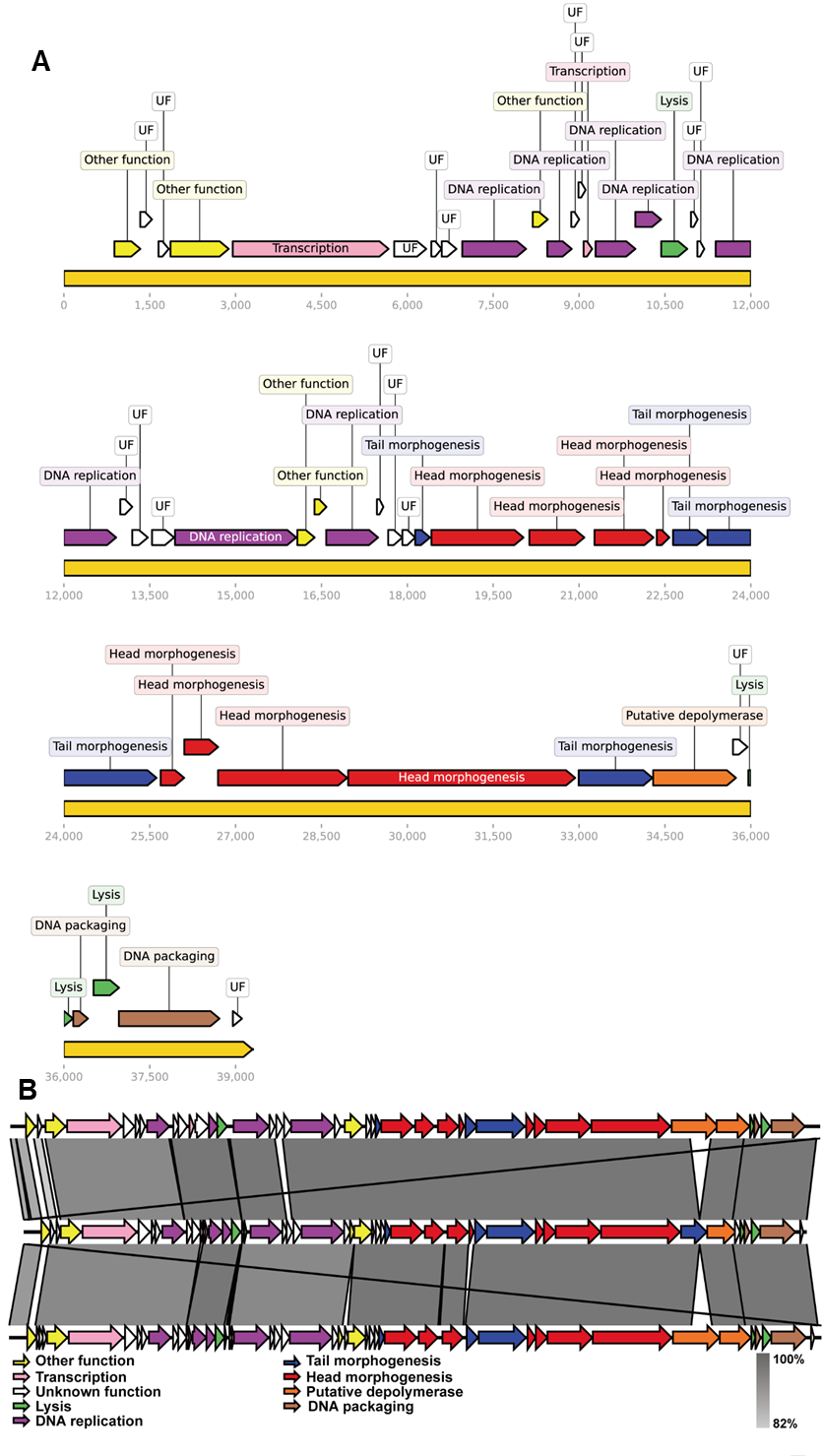


**Figure. S2** Genomic characterizations of H5. **(A)** Genome organization of phage H5. (**B)** Comparative analysis of phage Kpv 763, H5, and KP32. Different colored arrows represent predicted CDSs encoding different functions: red, lysis; gray, hypothetical protein (hp); orange, DNA replication, transcription, and metabolic function; green, phage structure; purple, DNA packaging.


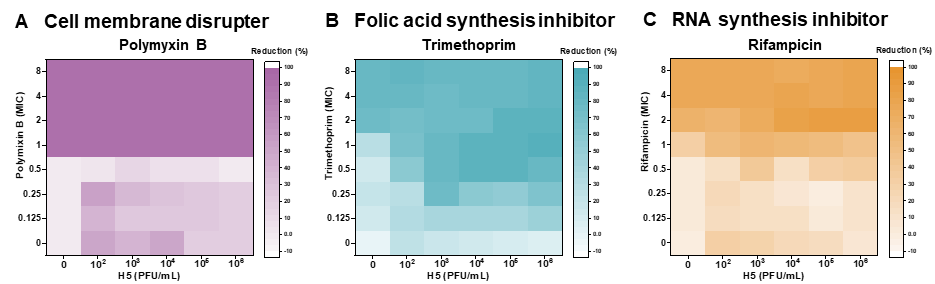


**Figure. S3** Effect of antibiotic class on phage-antibiotic synergy. Bacterial in log phase was inoculated into a 96-well plate coated with H5 and antibiotics, and the OD_600_ was measured every 10 min for a total of 24 h with shaking. The synergistic effect of H5 was estimated with: (**A**) polymyxin B; (**B**) trimethoprim; (**C**) rifampicin. Synograms (t = 24 h) represent the mean percentage reduction of each treatment from three biological replicates: Reduction (%) = [(OD _growth control_ - OD _treatment_)/OD _growth control_ ×100.


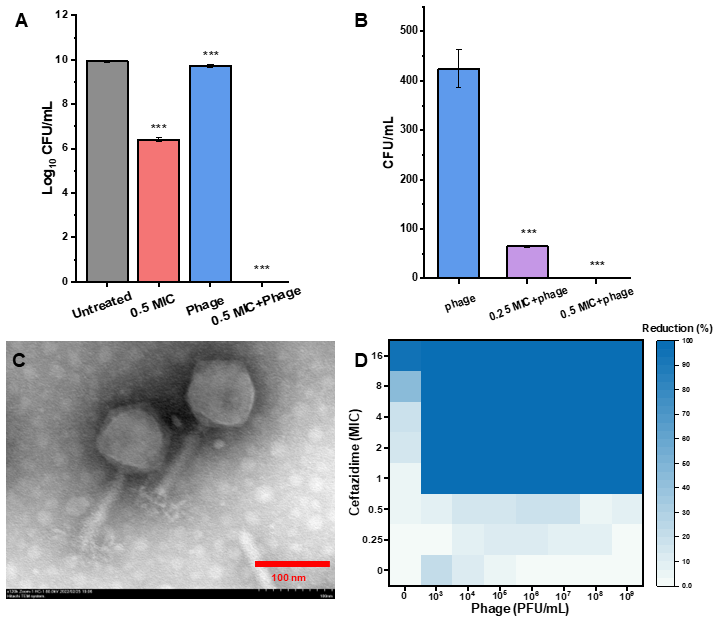


**Figure. S4** The synergy of phage and CAZ. Count statistics of wild type bacteria treated with H5 and H5-CAZ for 24 h (**A**) or 90 min (**B**). (**C)** The TEM images of phage B1. (**D)** The synergy of B1 and CAZ. Kpn 37485 in log-phase was inoculated into a 96-well plate coated with B1 and CAZ, and OD_600_ was measured every 10 min for a total of 24 h with shaking. Synograms (t = 24 h) represent the mean percentage reduction of each treatment from three biological replicates: Reduction (%) = [(OD _growth control_ – OD _treatment_)/OD _growth control_ ×100. Statistical analysis was performed using one-way ANOVAs tests. ^***^; *p* < 0.001.


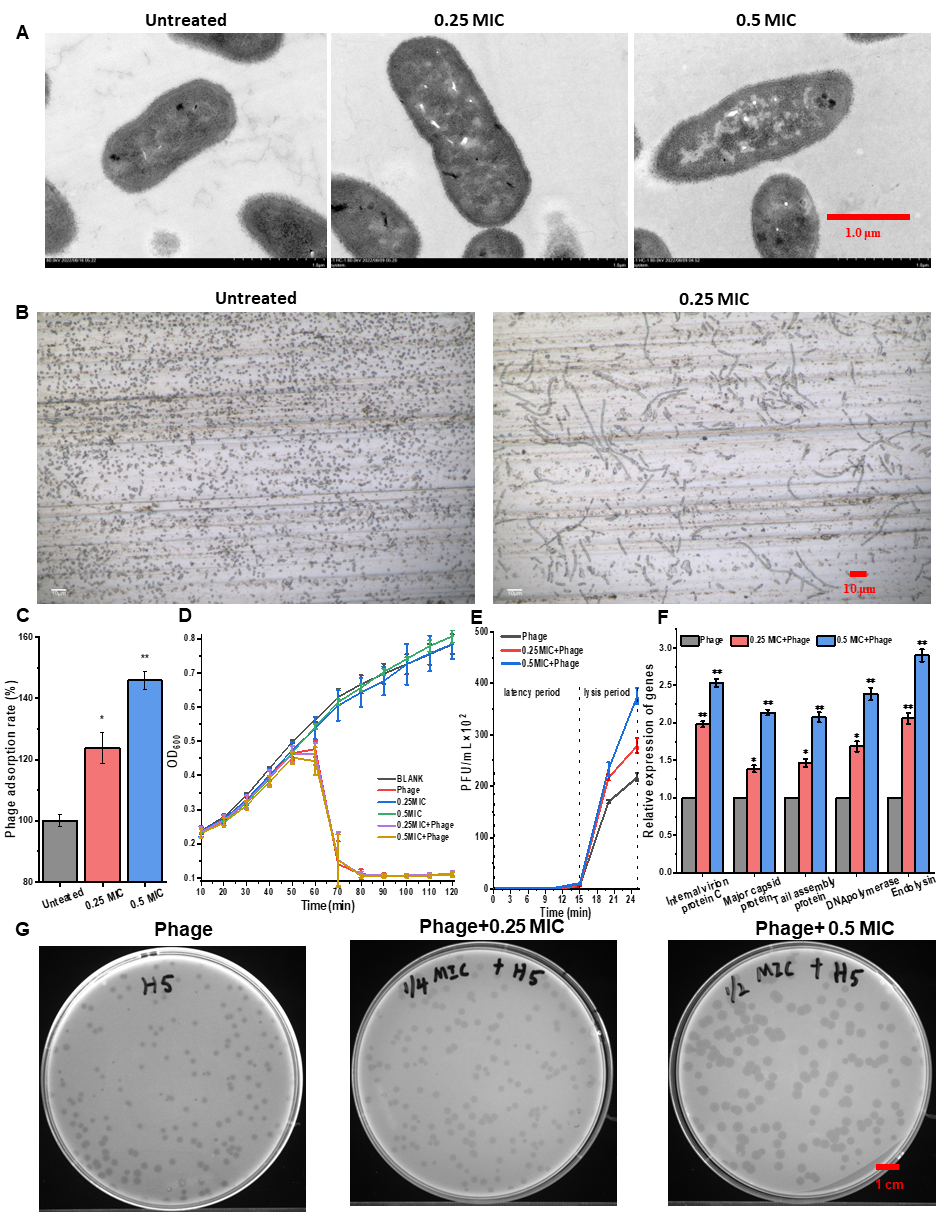


**Figure. S5** The synergy of H5 and CAZ. Effect of different concentrations of CAZ on cell size (**A-B**). (**C)** Effect of CAZ on phage adsorption. (**D)** Cell lysis assay of H5 *in vitro* for 2 h. (**E)** One-step growth curve of H5 in the presence of different doses of CAZ. (**F)** Effect of CAZ on the transcription levels of target genes in phage. (**G)** The synergistic effect of H5-CAZ on plaque size. Statistical analysis was performed using one-way ANOVAs tests. ^*^; *p* < 0.05 and ^**^; *p* < 0.01.


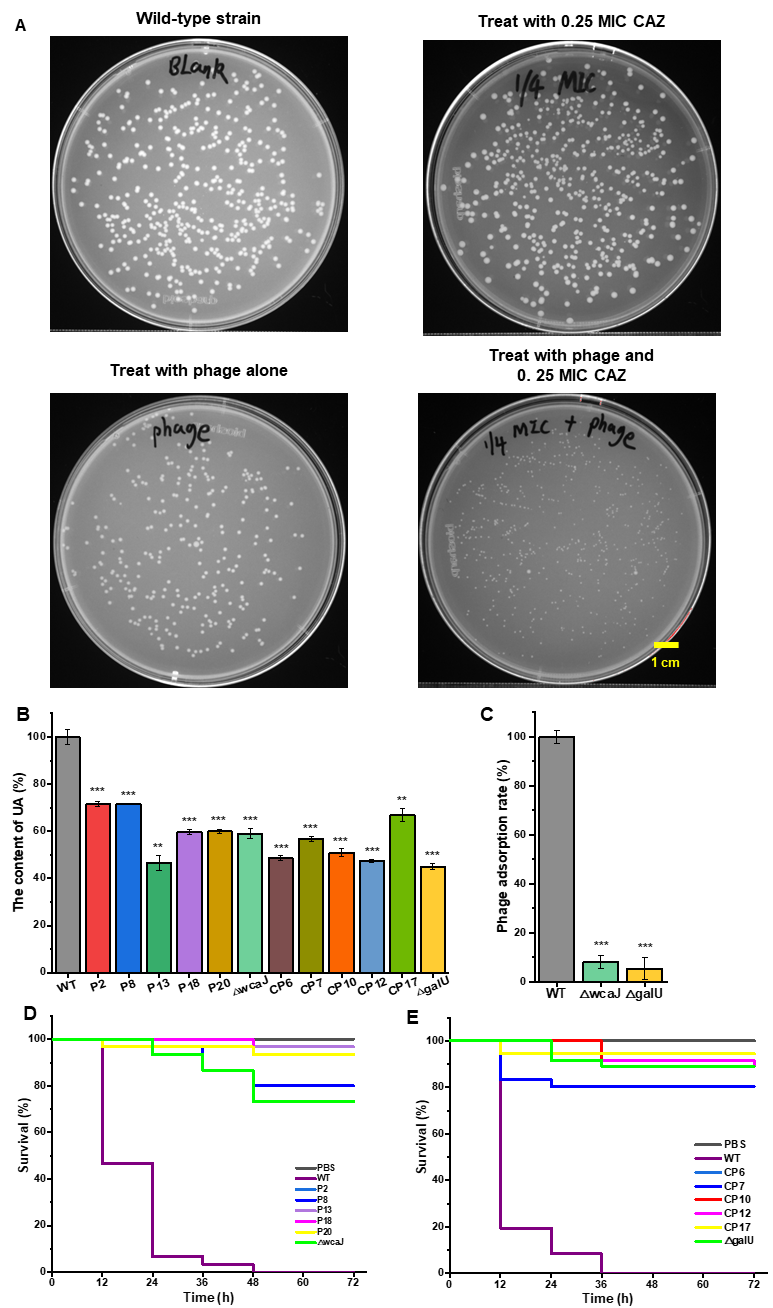


**Figure. S6** Characterization of mutants. **(A)** Morphological observation of single colony under different treatment conditions. (**B)** Uronic Acids (UA) content of WT and mutants. (**C)** Phage adsorption rate of 43816*△wcaJ* and 43816*△galU.* (**D-E)** Virulence of WT and mutant strains. Statistical analysis was performed using one-way ANOVAs tests. ^**^; *p* < 0.01 and ^***^; *p* < 0.001.


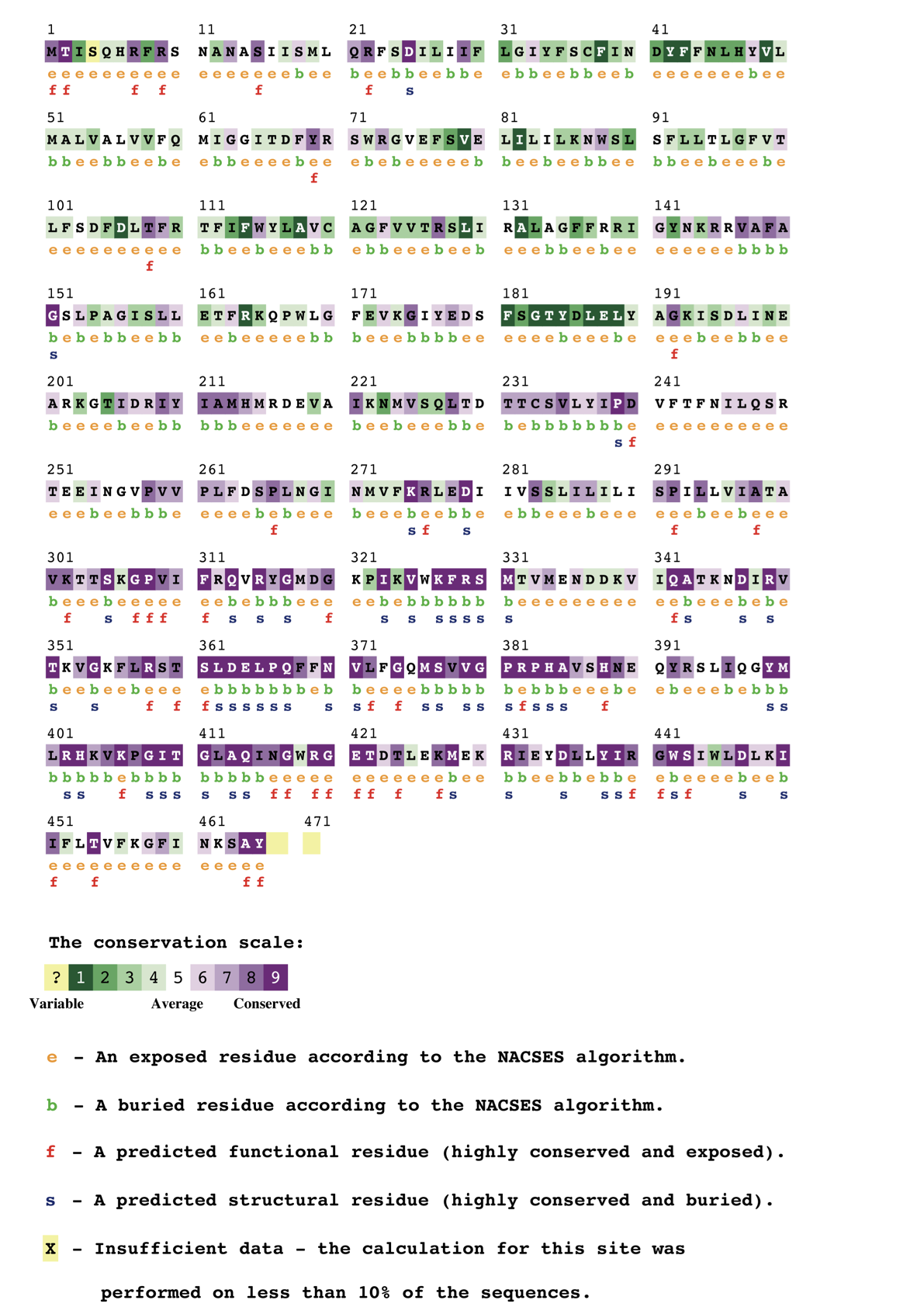


**Figure. S7** Sequence structure analysis of WcaJ.

**Figure. S8** Transcript levels of *ompK36* in mutant strains and 43816△*galU*. Statistical analysis was performed using one-way ANOVAs tests. ^*^; *p* < 0.05, ^**^; *p* < 0.01 and ^***^; *p* < 0.001.


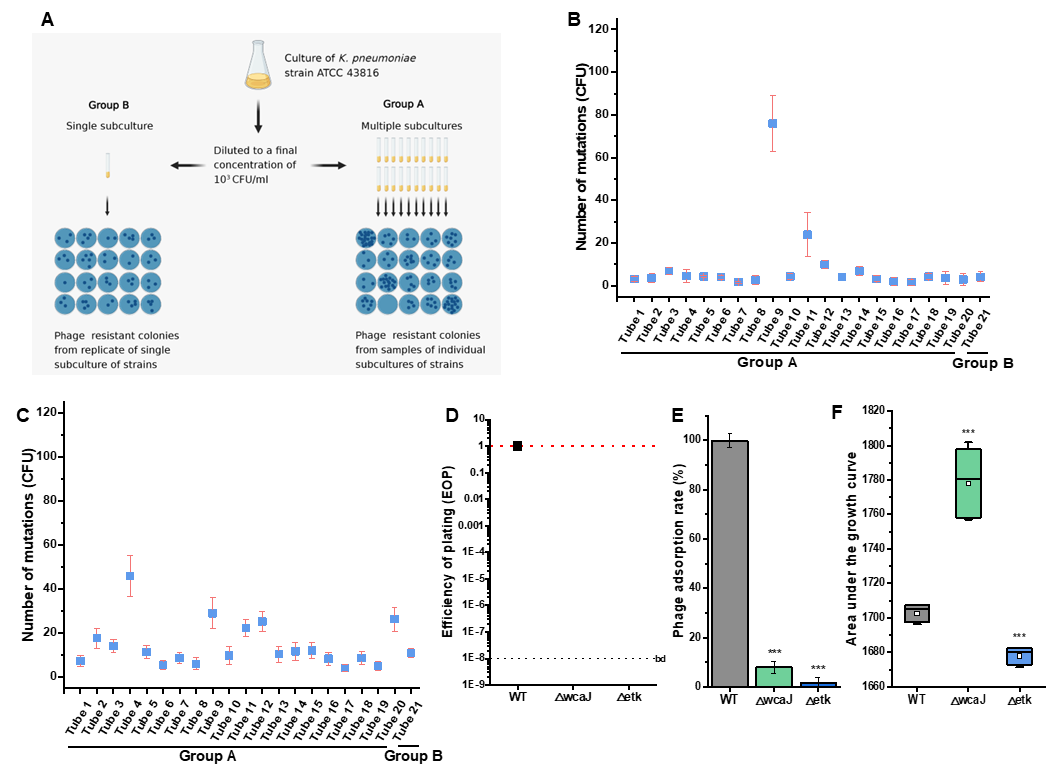


**Figure. S9** The fluctuation test under different conditions and the evolutionary advantage of *wcaJ* mutations compared to *etk*. **(A)** Flow chart and schematic of the fluctuation experiment set-up. (**B)** Count statistics of resistant colonies in the fluctuation test under phage treatment alone. (**C)** Count statistics of resistant colonies in the fluctuation test under phage-CAZ combination. Phage susceptibility (**D**), phage adsorption rate (**E**), and area under the growth curve (**F**) of WT, 43816*△wcaJ* and 43816*△etk.* Statistical analysis was performed using one-way ANOVAs tests. ^***^; *p* < 0.001.


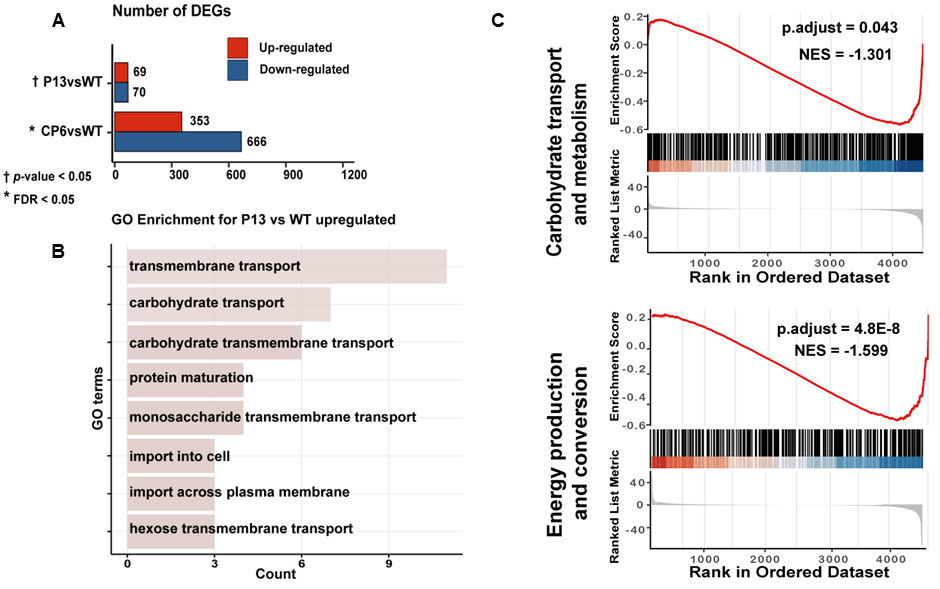


**Figure. S10** Transcriptome analysis of P13 and CP6. **(A)** The DEGs of P13 and CP6. (**B)** Go annotation analysis of up-regulated DEGs in P13. (**C)** Gene set enrichment analysis (GSEA) of down-regulated genes related to carbohydrate transport and metabolism and energy production and conversion in CP6.

**
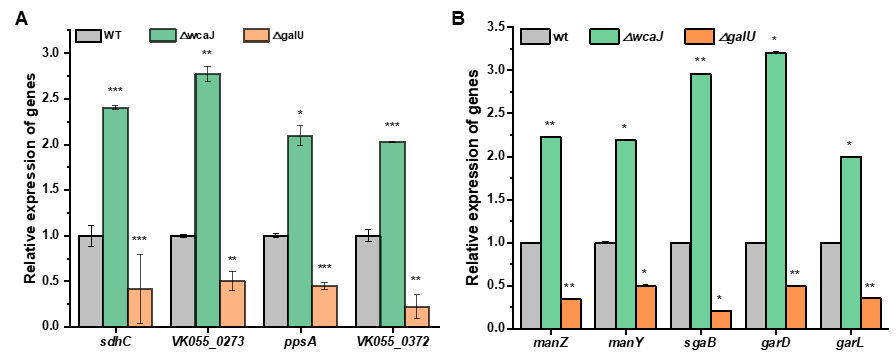
**

**Figure. S11** Transcript levels of carbon metabolism (**A**), phosphotransferase system (PTS) (**B**), and ascorbate and aldarate metabolism (**B**) genes in 43816△*wcaJ* and 43816△*galU*. Statistical analysis was performed using one-way ANOVAs tests. ^*^; *p* < 0.05, ^**^; *p* < 0.01 and ^***^; *p* < 0.001.


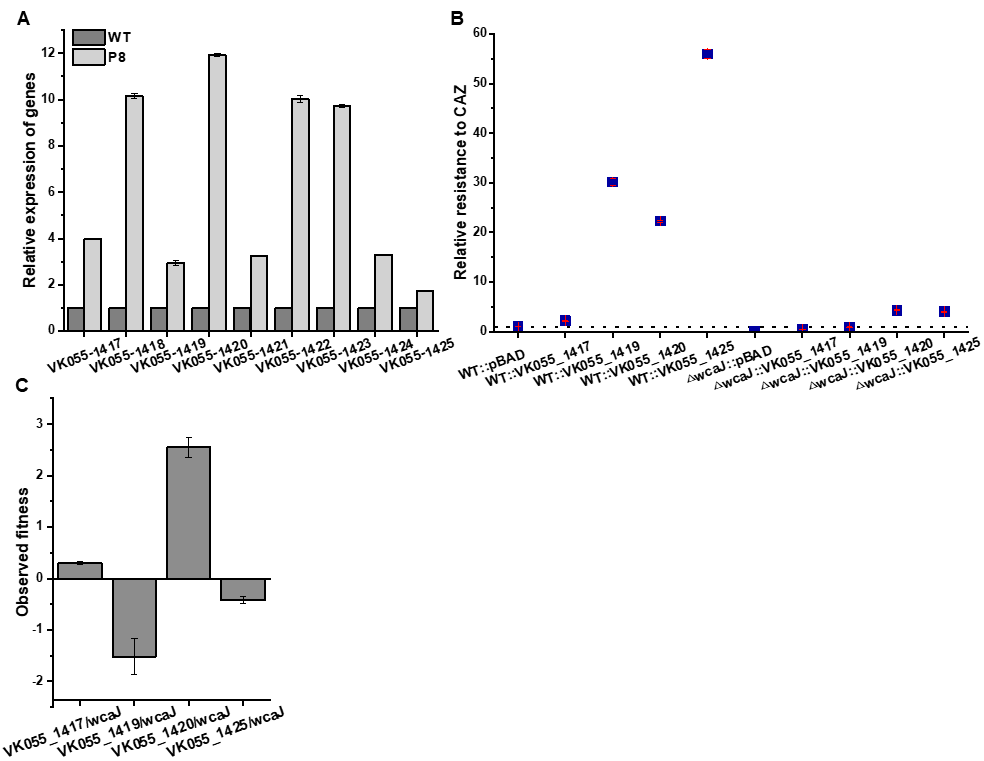


**Figure. S12** The mechanism involved in the switch from trade-off to trade-up in P8. (**A)** The transcript levels of genes inserted in P8. (**B)** The susceptibility to CAZ after overexpression of the insertion ORFs of P8 with known function in WT and 43816△*wcaJ*. (**C)** The observed fitness (epistasis (e)) of the double resistance genotypes. negative epistasis (e < 0); positive epistasis (e > 0). Susceptibility was defined as the ratio of the number of viable cells in the mutants compared to WT::pBAD strains under CAZ (0.25 mg/L) treatment.
